# Potential geographical distribution of *Cordyceps cicadae* and its two hosts in China under climate change

**DOI:** 10.3389/fmicb.2024.1519560

**Published:** 2025-01-15

**Authors:** Junyi Chen, Donglan He

**Affiliations:** Hubei Provincial Engineering and Technology Research Center for Resources and Utilization of Microbiology, College of Life Science, South-Central Minzu University, Wuhan, China

**Keywords:** *Cordyceps cicadae*, MaxEnt model, climate change, potential distribution, host

## Abstract

**Introduction:**

The fungus *Cordyceps cicadae* is both edible and medicinal.

**Methods:**

To acquire a thorough comprehension of its distribution in China, two host insects, *Macrosemia pieli* and *Platypleura kaempferi*, were selected as biological factors potentially associated with its distribution, the ENMTools program was utilized to ascertain the principal environmental factors affecting the distribution of potentially suitable habitats. The possible geographic distributions in the present as well as in the 2030s, 2050s, and 2070s were then predicted using the optimized MaxEnt model.

**Results:**

The primary environmental variables were soil pH, mean diurnal range, annual precipitation, precipitation seasonality, annual mean temperature and precipitation of the driest month. *C. cicadae* thrived on steep slopes. and some of which also significantly affect the distribution of its two hosts. Most of the suitable habitats of *C. cicadae* and *M. pieli* were currently found in the subtropical monsoon zone. The SSP126, SSP370, and SSP585 scenarios were associated with positive, stable, and unfavorable impacts on the extent of suitable habitats for *C. cicadae*, respectively, and the suitability of *P. kaempferi* decreased under three different conditions. The expansion of the *C. cicadae* was observed in provinces bordering the middle and lower reaches of the Yellow River, as well as in Zhanjiang, Guangdong Province, and northern Yunnan Province. Conversely, its habitat contraction was mainly found in western Guangdong, southern Guangxi, northern Hainan, southwestern Yunnan, and areas bordering eastern Sichuan. The shared contraction regions with its two hosts were primarily located in western Guangdong, southern Guangxi, and southern Sichuan. Moreover, the future centroids were found at higher elevations than the present ones in the provinces of Jiangxi and Hunan.

**Discussion:**

In light of climate change, this research held significance for the conservation and sustainable utilization of *C. cicadae*.

## 1 Introduction

Our climate has changed and will continue to evolve (Yuan et al., 2024). A series of studies have shown that many plants and animals are facing serious challenges to their survival due to climate warming, leading to temporal and spatial shifts in their distribution ranges (Guo et al., 2017), Climate warming affects the survival of species by limiting their dispersal rates (Harrison, 2020), ultimately leading to range reductions (Harsch and HilleRisLambers, 2016)and accelerated extinctions (Sandel et al., 2011). The recognized valuable macrofungi are also being compelled by climate change to shift their geographic ranges (Schoenenberger-Arnaiz et al., 2017). A considerable number of economically valuable mushrooms undergo a decrease since the prolongation of the phenology (Thomas and Büntgen, 2019). Global warming will significantly increase during the next 20 years (Klutse et al., 2021). Therefore, it is vital to focus on macrofungi, particularly the high medicinal and food value species, to assess the possible effects of climate change on their range, and to develop a management plan to preserve the variety and wild resources of macrofungi.

*Cordyceps cicadae* was approved as a medicinal product by the Chinese Food and Drug Administration (CFDA) in 2021 (Zhao et al., 2022). *C. cicadae* is a parasitic strain of *Paecilomyces cicadae* that is developed on cicadas. *C. cicadae* was known to have anti-inflammatory, hypoglycemic, anticancer, and renal and eye protective effects (Sun et al., 2017; Zhu et al., 2014; Deng et al., 2020; Sun et al., 2021; Yang et al., 2024). Numerous useful secondary metabolites have also been shown to be present in it, including the pancreatic lipase inhibitor cerebroside, cardiovascular-protecting N6-(2-hydroxyethyl) adenosine and the anti-aging saponin A from *Nicotiana tabacum* (Dong et al., 2024).

Furthermore, the primary source of data on the distribution of *C. cicadae* in China has been field surveys (Huang et al., 2021; Zhao et al., 2021; Chen et al., 2023; Li et al., 2023). Examining the diversity of the genus *Cordyceps* in a particular region was the main objective. The relationship between their distribution and environmental conditions has been rarely studied. Only in Sichuan Province and Zhejiang Province’s Anji County did research establish a connection between ecological factors and the present geographic distribution (Huang et al., 2021; Li et al., 2023). Interestingly, only one study utilizing species distribution models projected the distribution of *C. cicadae* in China (Zhang et al., 2022). Through modeling based on temperature and precipitation, it found important environmental variables, such as the minimum temperature of the coldest month, the precipitation of the coldest quarter, and isothermality. Furthermore, the study discovered a significant loss in suitable habitats by using a future climatic scenario. This research not only made the first prediction of the distribution of *C. cicadae* in China by highlighting the critical influences of temperature and precipitation on the habitats, but it also increased awareness of the need to safeguard ecological environment of *C. cicadae*. However, the narrow range of climate scenarios and models was insufficient to minimize the uncertainty in results arising from changes in climate models and to take into account the effects of shifting future growth patterns on the regional distribution. Furthermore, it omitted to address the connections that exist between the incidence of *C. cicadae* and other environmental factors. For instance, *C. cicadae* preferred to live in acidic soils (pH of 5.9) that had a high concentration of organic matter and total nitrogen. In comparison to locations where *C. cicadae* did not develop, these soils had significantly lower amounts of accessible phosphorus, available potassium, and total phosphorus (Huang et al., 2021). Topographic investigations reveal a strong relationship between slope and *C. cicadae* growth density in Sichuan Province (Li et al., 2023). As slope steepness increases, there was a first increase in growth density and then a subsequent decrease. A link was also seen in the vegetation composition, with *C. cicadae* preferring to develop in woodlands with *Phyllostachys edulis* and tea plants (Huang et al., 2021). Additionally, the distribution of host insects deserves consideration. Although previous studies have primarily focused on how fungi infect and control their hosts, The relationship between the geographic distribution of fungi and their hosts has rarely been studied, the relationship between the geographic distribution of fungi and their hosts has been rarely explored. To accurately address the detrimental effects of climate change, species distribution predictions must consider a broader range of factors, climate models, and scenarios.

In China, the *Platypleura kaempferi* and the *Macrosemia pieli* were common host insects of the *C. cicadae*. Our research utilized the distribution records and environmental factors of *C. cicadae, P. kaempferito* and *M. pieli* to estimate probable suitable habitats for the species under both present and future climatic scenarios through an optimized the Maximum Entropy Model (MaxEnt). From this study, we can: (1) identify environmental factors that influence species distribution; (2) project the potential geographic distribution range of *C. cicadae* and its two hosts under the current climate scenarios; (3) anticipate changes in the areas of various classes of suitable habitats under the future climate scenarios; (4) predict changes in the spatial and temporal distribution of centroids of the suitable habitats under the future climate scenarios. This study was the first to more fully investigate the effects of environmental and biological variables on the distribution of *C. cicadae* and to evaluate the implications of multiple future temperature scenarios on the suitability areas and centroids of *C. cicadae* and its two hosts. The knowledge gathered from this work considerably benefited in the genetic selection of *C. cicadae*, habitat design, bionic cultivation, and conservation of *Cordyceps* resources.

## 2 Materials and methods

### 2.1 Software

This study utilized MaxEnt (version 3.4.4) (Phillips and Dudík, 2008), ENMTools (version 1.3) (Warren et al., 2010), the R programming environment (version 4.3.3) (Team, 2014) to assess species distribution data and environmental parameters and applied ArcGIS (version 10.8) (Dai et al., 2023) for mapping. The MaxEnt employed the principle of maximum entropy to analyze the relationship between environmental factors and species presence, and to predict species distribution under future climate scenarios (Hernandez et al., 2006; Fang et al., 2024). A program called ENMTools was utilized to evaluate species niches, characterize ecological tolerance, and evaluate habitat suitability. A collection of objects, variables, functions, and other elements used for statistical computing and graphics is known as the R programming environment. A geographic information system program called ArcGIS makes it possible to create maps, manage spatial data, and integrate spatial information.

### 2.2 Data

#### 2.2.1 Distribution records

**Figure 1:**
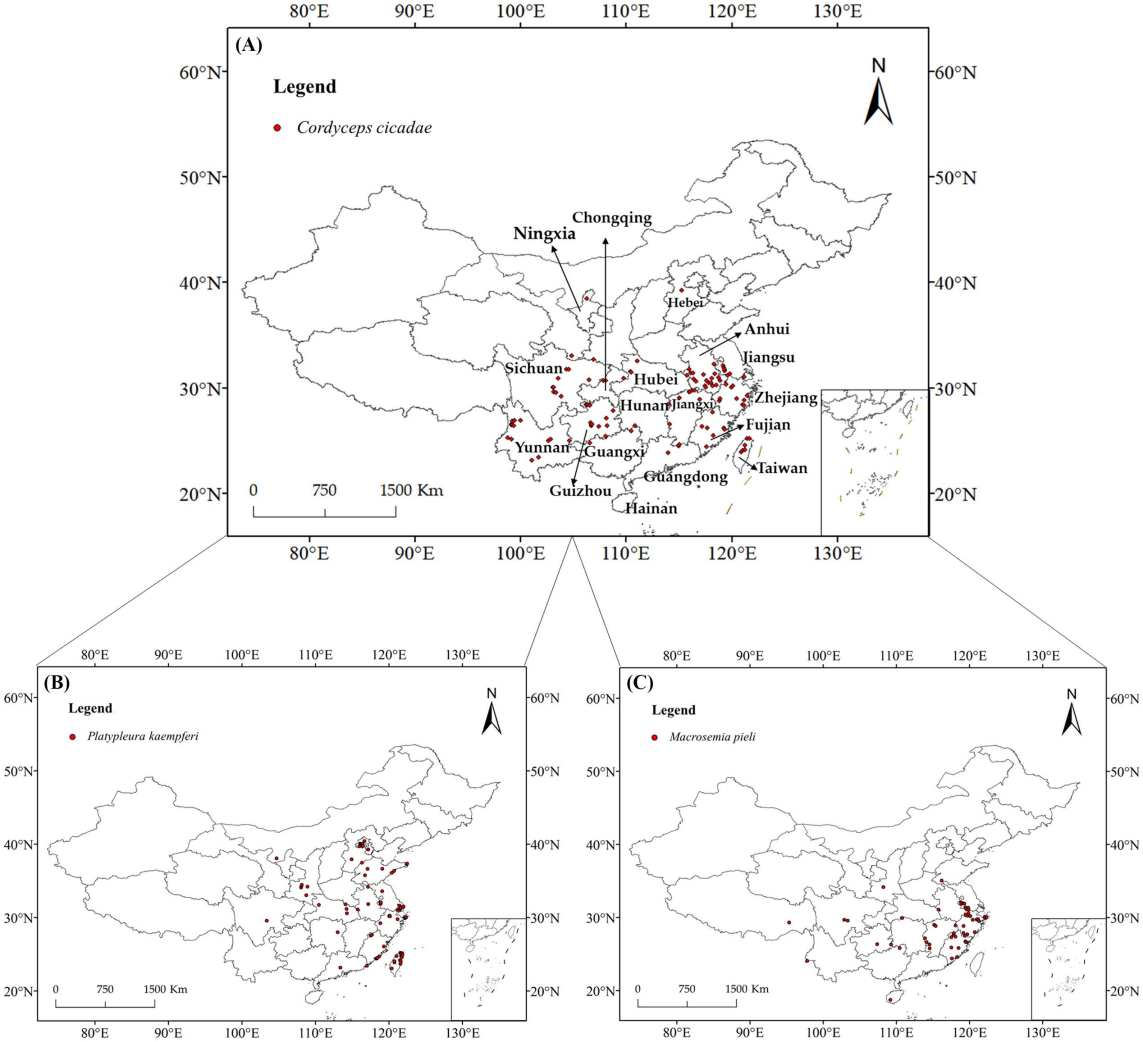
Distribution information of *C. cicadae* and its two host insects in China: **(A)** *C. cicadae*; **(B)** *P. kaempferi*; **(C)** *M. pieli*.

The Global Biodiversity Information Facility (GBIF^1^), the China Field Herbarium (CFH^2^), the Mycological Herbarium of the Institute of Microbiology of the Chinese Academy of Sciences,^3^ the National Specimen Information Infrastructure (NSII),^4^ the Taiwan Biodiversity Information Facility (TaiBIF)^5^ and related literature (Supplementary Data Sheet 1) on the distributional data of *C. cicadae*, *P. kaempferi*, and *M. pieli*, were gathered for our study. In addition, we obtained two additional distribution points in July–August 2023 during a field survey. The coordinates were calibrated using the OvitalMapV9.0 program,^6^ and duplicate locations with straight-line distances of less than 10 km were eliminated to prevent overfitting. Ultimately, 114 *C. cicadae* distribution points, 86 *P. kaempferi* distribution points, 55 *M. pieli* distribution points, and 131 *O. maculaticollis* distribution points were gathered (Figure 1).

#### 2.2.2 Environmental variables

A total of 28 environmental variables—soil, terrain, vegetation, and climate—potentially influencing the growth and distribution of *C. cicadae* were identified and categorized into four categories for the study (Supplementary Table S1). Soil parameters were sourced from the Chinese Soil Organic Matter Data set (1980s) supplied by the National Tibetan Plateau Scientific Data Center^7^ (Shangguan et al., 2013), encompassing pH, soil organic matter (SOM), total phosphorus (TP), total nitrogen (TN), available phosphorus (AP), and available potassium (AK). Topographic factors were derived from the Shuttle Radar Topography Mission Digital Elevation Model (SRTMDEM) 90 m resolution elevation data (2000) available on the Geospatial Data Cloud,^8^ elevation and slope were extracted from ArcGIS 10.8 software. Vegetation factors, particularly vegetation types, were obtained provided by the 1-million vegetation type spatial distribution dataset (2001) from the Chinese Academy of Sciences’ Resource and Environmental Science Data Center.^9^ Climatic variables were obtained from the WorldClim database (version 2.1),^10^ encompassing 19 climatic parameters from 1970 to 2000. The spatial extent was masked to China through the extraction analysis tool in ArcGIS. The final data was stored in “ASC” format after all environmental variables were resampled to a consistent resolution of 30 arc-seconds and standardized to the geographic coordinate system of “WGS 1984.”

The Climate Model Intercomparison Project (CMIP) is a global climate model jointly launched by the United Nations Intergovernmental Panel on Climate Change (IPCC) (Deepa et al., 2024), In comparison to CMIP5, CMIP6 provided substantial enhancements in future climate forecasts (Yang et al., 2021). This study selected three Global Climate Models (GCMs) from the Coupled Model Intercomparison Project Phase 6 (CMIP6): the Beijing Climate Center Climate System Model version 2 Medium Resolution (BCC-CSM2-MR), the Max Planck Institute Earth System Model (MPI-ESM1-2-HR) and the EC-Earth3 Earth System Model (EC-Earth3-Veg) (Yang et al., 2023). The decades of 2050s (2041–2060), the 2070s (2061–2080), and the 2030s (2021–2040) were utilized to ascertain future climate variables. We employed three Shared Socioeconomic Pathways (SSPs): SSP126, SSP370, and SSP585 to model potential future climatic scenarios. SSP126 illustrated a low-radiative forcing scenario with minimal carbon emissions and represented a future characterized by limited development pressure and sustainability. Conversely, SSP585 relied on fossil fuels, indicating a future characterized by substantial emissions and unsustainable development under elevated radiation forcing. The SSP370 model forecast a moderate future scenario characterized by intermediate levels of land utilization and carbon emissions (Deepa et al., 2024). ArcGIS software was employed to ascertain the mean outputs of all GCMs to mitigate result bias. This approach generated nine datasets (comprising three temporal intervals and three climatic scenarios) intended for subsequent species distribution modeling.

### 2.3 Methods

#### 2.3.1 Screening of environment variables

**Figure 2:**
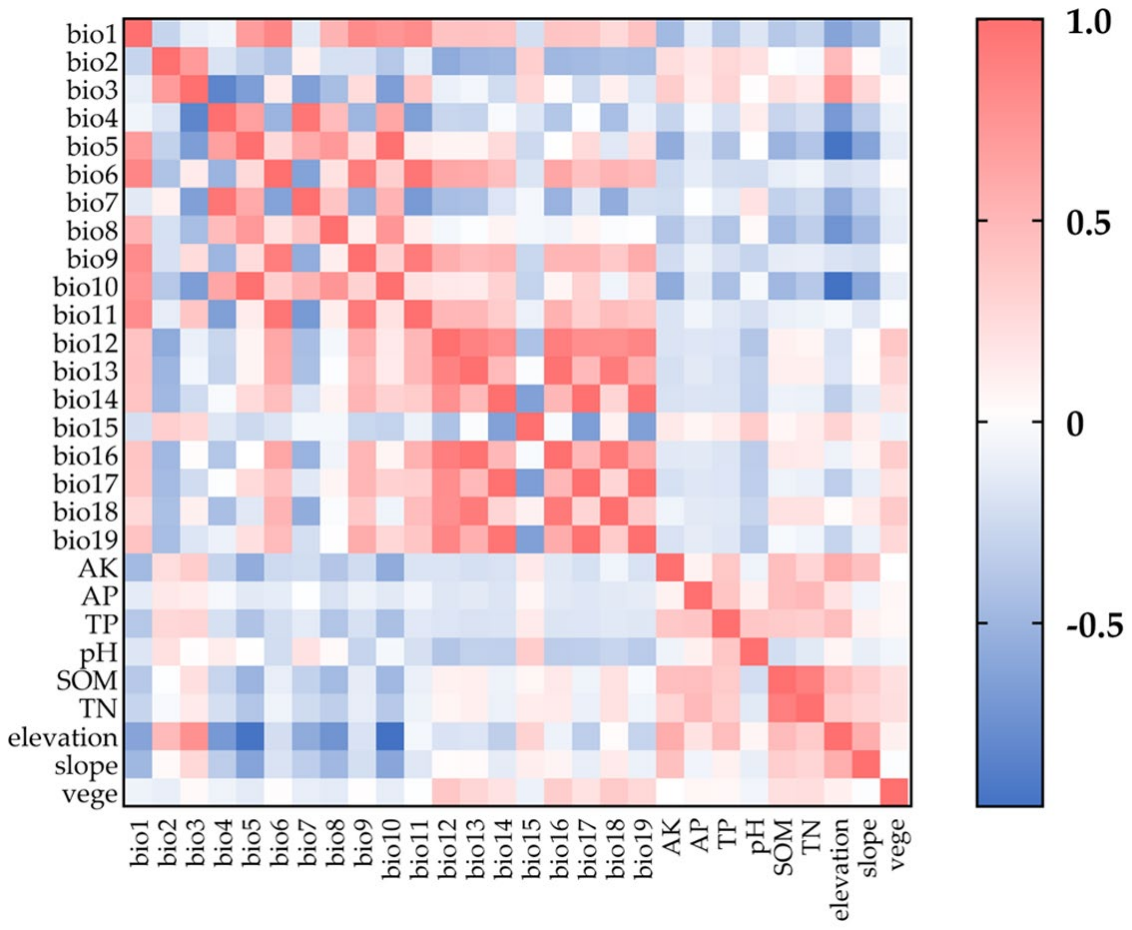
The correlation heat map of environmental variables.

We analyzed the correlations among environmental data, illustrated in a heatmap (Figure 2), utilizing the Pearson method in ENMTools. MaxEnt was employed to import the data and execute 10 iterations of each (Xian et al., 2023). We eliminated one of any two variables exhibiting a Pearson correlation coefficient |*r*| ≥ 0.8 to mitigate redundancy caused by autocorrelation and multicollinearity, retaining the variable that contributed more significantly to the modeling. Ultimately, we modeled the geographical distribution of *C. cicadae*, *P. kaempferi* and *M. pieli* utilizing 14 environmental variables (Supplementary Tables S2–S4).

#### 2.3.2 MaxEnt model calibration and evaluation

The feature classes (FC) and regularization multiplier (RM) parameters were pivotal in the MaxEnt model, substantially affecting the output outcomes (Ahmadi et al., 2023). The MaxEnt model correlates feature classes with empirical data, however the regularization multiplier imposes supplementary restrictions to regulate model complexity. Researchers can calibrate and assess the MaxEnt model by modifying the values of feature classes and regularization multipliers, thereby improving its accuracy. In the calibration procedure, “l,” “q,” “p,” “t,” and “h” were five unique feature parameters, denoting linear, quadratic, product, threshold, and hinge, respectively. The parameters were amalgamated to create six feature classes: “l,” “lq,” “h,” “lqh,” “lqhp,” and “lqhpt.” The RM values were established at 20 distinct levels, spanning from 0.5 to 10, with increments of 0.5. This study used the Kuenm package in R programming environment to screen these 120 models (Xue et al., 2024).

#### 2.3.3 Analysis of environmental factors

The jackknife test is a statistical technique used to assess the weight of each environmental variable in a model. To determine the primary environmental factors affecting the distribution of potentially suitable habitats, the jackknife test results and the contribution rate (Supplementary Table S2) were integrated and analyzed. The single-factor environmental response curve was utilized to ascertain the threshold value of the environmental variable.

#### 2.3.4 Analysis of different classes of suitable habitats

##### 2.3.4.1 Classification of suitable habitats

We utilized the prediction outcomes from various future climatic situations with the present scenario for mapping purposes. Utilizing the maximum test sensitivity plus specificity (MTSPS) threshold in conjunction with the output omission rate of the MaxEnt model and species distribution probability P, the suitable areas were classified into unsuitable habitats (MTSPS value ≤ *p* ≤ 0.4), poorly-suitable habitats (0.4 ≤ *p* ≤ 0.6), moderately-suitable habitats (0.6 ≤ *p* ≤ 0.8), and highly-suitable habitats (0.8 ≤ *p* ≤ 1.0).

##### 2.3.4.2 Calculation of the area of different classes of suitable habitats

An ArcGIS map comprised several pixels, and the ratio of pixels in each region to the total pixel count on the map represented the area proportion of each region. The subsequent formula was employed to calculate the area of suitable regions at different levels:

$\begin{array}{l} \mathrm{Area}=\left(\mathrm{Proportion of suitable area for different levels}\right)\times \\ \mathrm{total land area of China}\;\left({\mathrm{km}}^{2}\right)\text{.} \end{array}$

#### 2.3.5 Changes of spatiotemporal and centroid in suitable habitats

The SDM toolbox^11^ was utilized to do binary processing of the suitable habitats, designating areas with thresholds below MTSPS as unsuitable habitats, and areas with distribution probabilities equal to or beyond MTSPS as acceptable habitats. In the result file, “-1” denoted the expansion zone of acceptable habitats, “0” signified unsuitable habitats, “1” represented the retention region of suitable habitats, and “2” indicated the contraction region of suitable habitats. Trends in the appropriate habitats of *C. cicadae* and its two hosts were assessed for present and prospective situations. Simultaneously, math tools were employed to overlay the contraction areas of the *C. cicadae* and its two hosts.

The center of mass position, indicating the overall spatial distribution of suitable habitats for *C. cicadae*, was ascertained utilizing the SDM tool. The MTSPS value served as a criterion to delineate total value acceptable and unsuitable habitats. This configuration reflected the transmission of *C. cicadae* and its two hosts in both general suitable habitats and habitats moderately to highly suited.

## 3 Results

### 3.1 MaxEnt model optimization and accuracy evaluation

**Figure 3:**
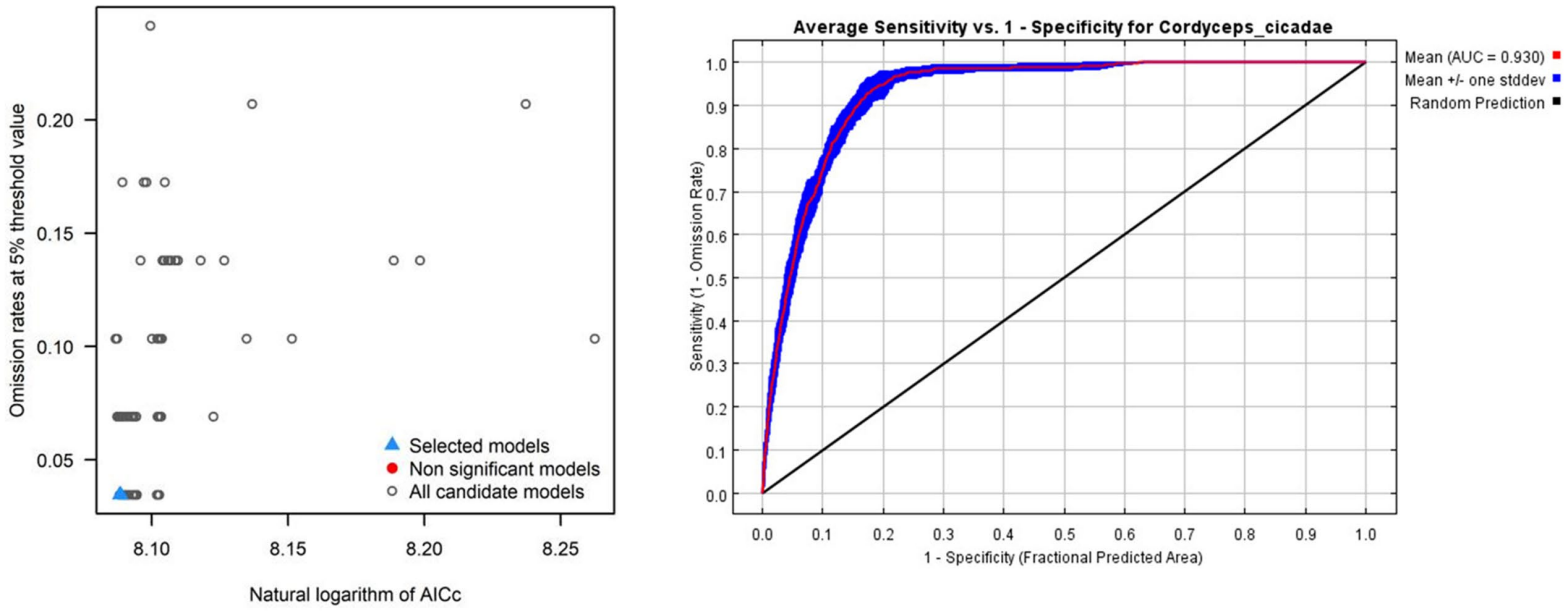
MaxEnt model parameter calibration results of *C. cicadae*: **(A)** Results of the selection of the optimal models; **(B)** ROC curves and AUC values of MaxEnt model prediction results. The black line represented random prediction, whereas the red curve represented training data.

The best model must satisfy three criteria: (1) omission rate less than 5% (2) statistically significant (3) less than two delta AICc values (Cobos et al., 2019). The model for *C. cicadae* met these criteria when executed with an RM of 4.5 and FCs of “lqph,” demonstrating an omission rate of 0.034 and a delta AICc value of 0 (Figure 3A). It was also statistically significant. Meanwhile, the optimized models for *P. kaempferi* (Supplementary Figure S1A) and *M. pieli* (Supplementary Figure S1C) satisfied the criteria with RM values of 0.5 and 6.5, and FC values of “lq” and “lqh” respectively. The analysis indicated that the optimal model exhibited a reduced level of overfitting compared to the default model, leading to enhanced output accuracy. The default model exhibited a delta AICc value of 624.723. Additionally, 0.930 was shown to be the optimum model’s average AUC value for *C. cicadae* (Figure 3B), 0.929, 0.926 were shown to be the optimum model’s average AUC value for *P. kaempferi* (Supplementary Figure S1B) and *M. pieli* (Supplementary Figure S1D). This indicated that the optimal MaxEnt model accurately predicted the potential distribution of *C. cicadae* and its two hosts in China and will be employed in future research.

### 3.2 Dominant environmental variables

**Figure 4:**
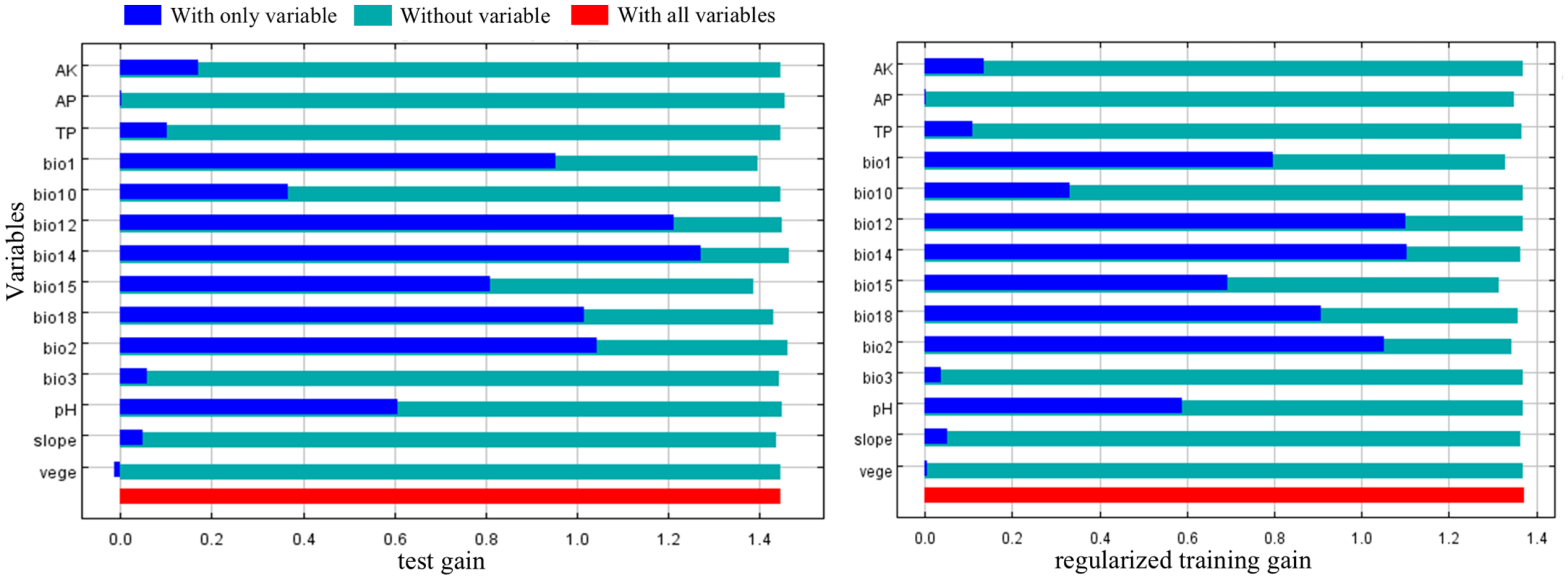
The jackknife method highlighting the importance of environmental variables for *C. cicadae.*

The precipitation factors bio12, bio14 and bio15 accounted for the highest cumulative contribution rate of 88.1% in the research findings, underscoring the significant influence of precipitation on species dispersion. Bio14 emerged as the most significant component, exhibiting the highest replacement importance and contribution rate among these elements, and provided the most unique data for modeling *C. cicadae* distribution (Figure 4). In addition to precipitation, soil pH and temperature factors (bio1, bio2) were also significant. In summary, bio14, bio12, bio1, bio15, bio2, and pH were the principal parameters influencing the distribution of *C. cicadae*.

**Figure 5:**
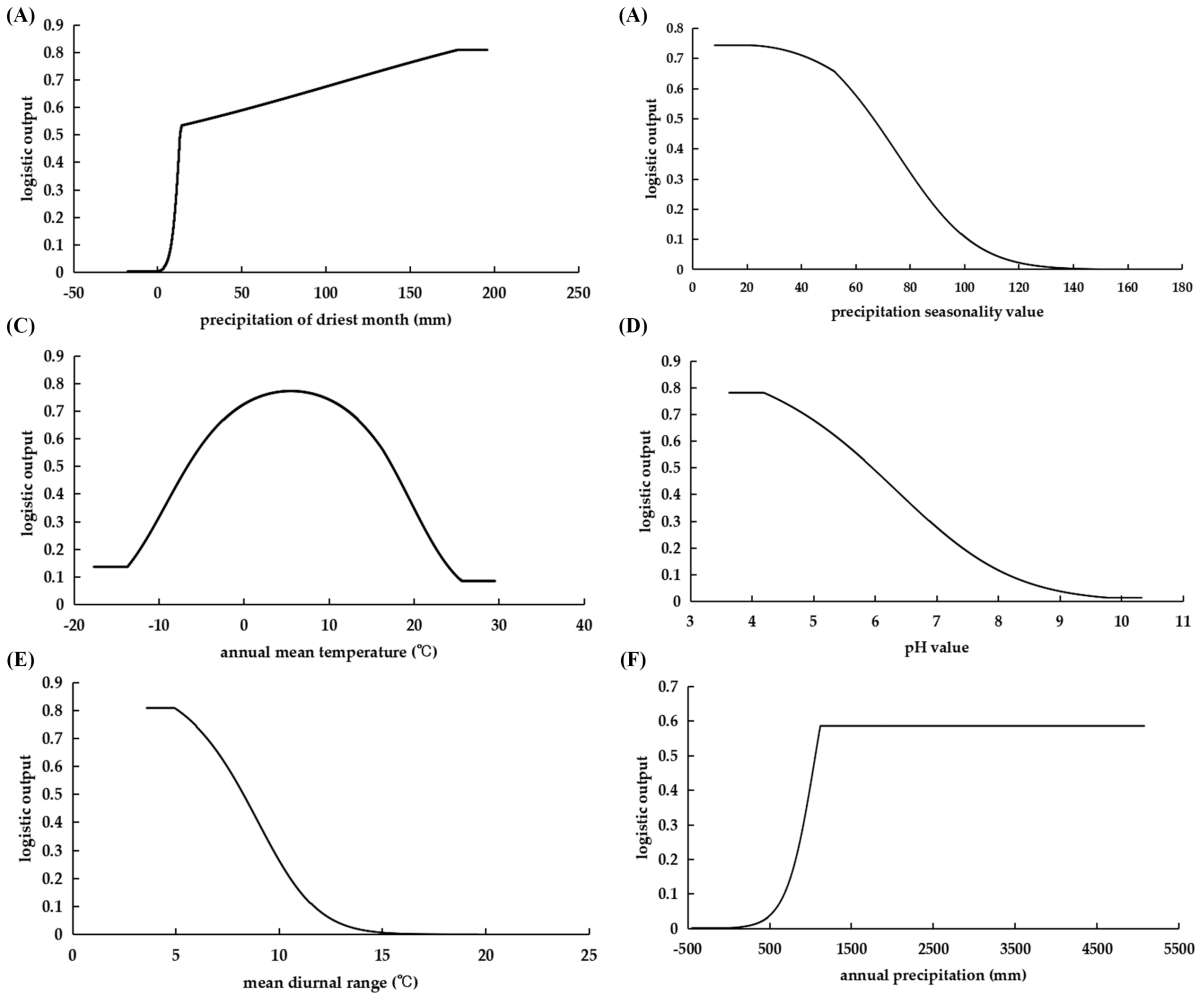
Response curves of the probability of *C. cicadae* presence to dominant environmental variables: **(A)** bio14; **(B)** bio15; **(C)** bio1; **(D)** pH; **(E)** bio2; **(F)** bio12.

Environmental variable thresholds suitable for species dispersal were frequently delineated with a presence probability of 0.5 or above. The correlation between various environmental variables and species distribution probabilities was demonstrated by univariate environmental response curves (Figure 5). The habitats conducive to the proliferation of *C. cicadae* exhibited a minimum of 13.81 mm of precipitation during the driest month and an annual rainfall exceeding 1065.16 mm. Increased rainfall during the warmest season improved its survival suitability, whereas higher seasonal precipitation coefficients diminished it. The optimal annual temperature range for *C. cicadae* was −6.61°C to 17.43°C, accompanied by a daily temperature fluctuation of 3.58°C to 8.27°C. For soils, the ideal pH range was 3.63–5.94, with 3.63 being the best.

For *P. kaempferi*, the primary environmental factors identified were elevation, bio2, bio14, bio15, bio8, and bio6, with elevation contributing the most significantly. The suitability of *P. kaempferi* decreased as elevation increased, with an optimal elevation range of less than 398.85 m. Bio2 provided the most unique information, indicating that the suitability of *P. kaempferi* declined as the diurnal temperature range increased, with an ideal range between 3.73°C and 8.79°C, similar to *C. cicadae*. Its precipitation requirement during the driest month was greater than that of the *C. cicadae* (Supplementary Figure S2).

For *M. pieli*, the dominant environmental factors were bio14, bio6, bio15, pH, bio10, and bio9, with bio14 contributing the most significantly. The optimal range for bio14 was above 16.93 mm, aligning closely with the *C. cicadae*. Bio2 provided the most unique information, indicating that the min temperature of coldest month should range between −6.05°C and 9.68°C. The trends of bio15 and pH were consistent with those of *C. cicadae*, with an ideal pH range of 3.67–6.1, where 3.67 was considered optimal. Furthermore, the mean temperature of the warmest quarter and the mean temperature of the driest quarter should be above 22.60°C and 2.82°C, respectively (Supplementary Figure S2).

### 3.3 Analysis of topographic factor

**Figure 6:**
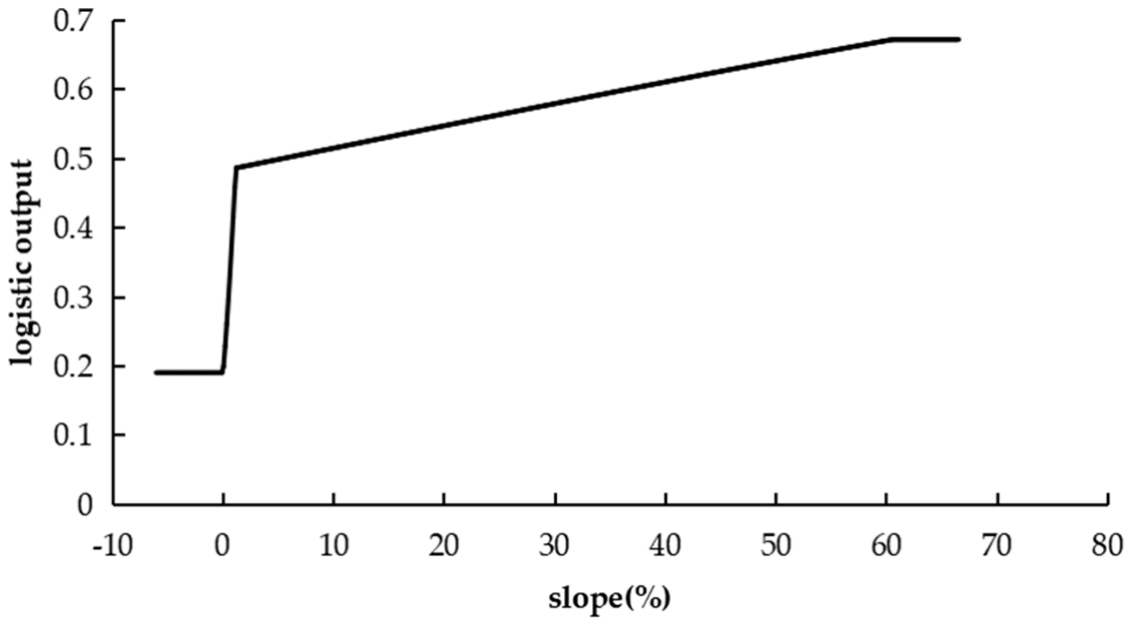
Response curves indicating the probability of *C. cicadae* presence relative to the slope.

Topographic factors were more significant to the model distribution than vegetation type, yet less than climate and soil. The suitability of *C. cicadae* escalated with slope, indicating a preference for elevated, more perilous environment (Figure 6).

### 3.4 Prediction of the suitable habitat of *Cordyceps cicadae* and its two hosts in China

#### 3.4.1 Potential geographical distributions under the current climate

**Figure 7:**
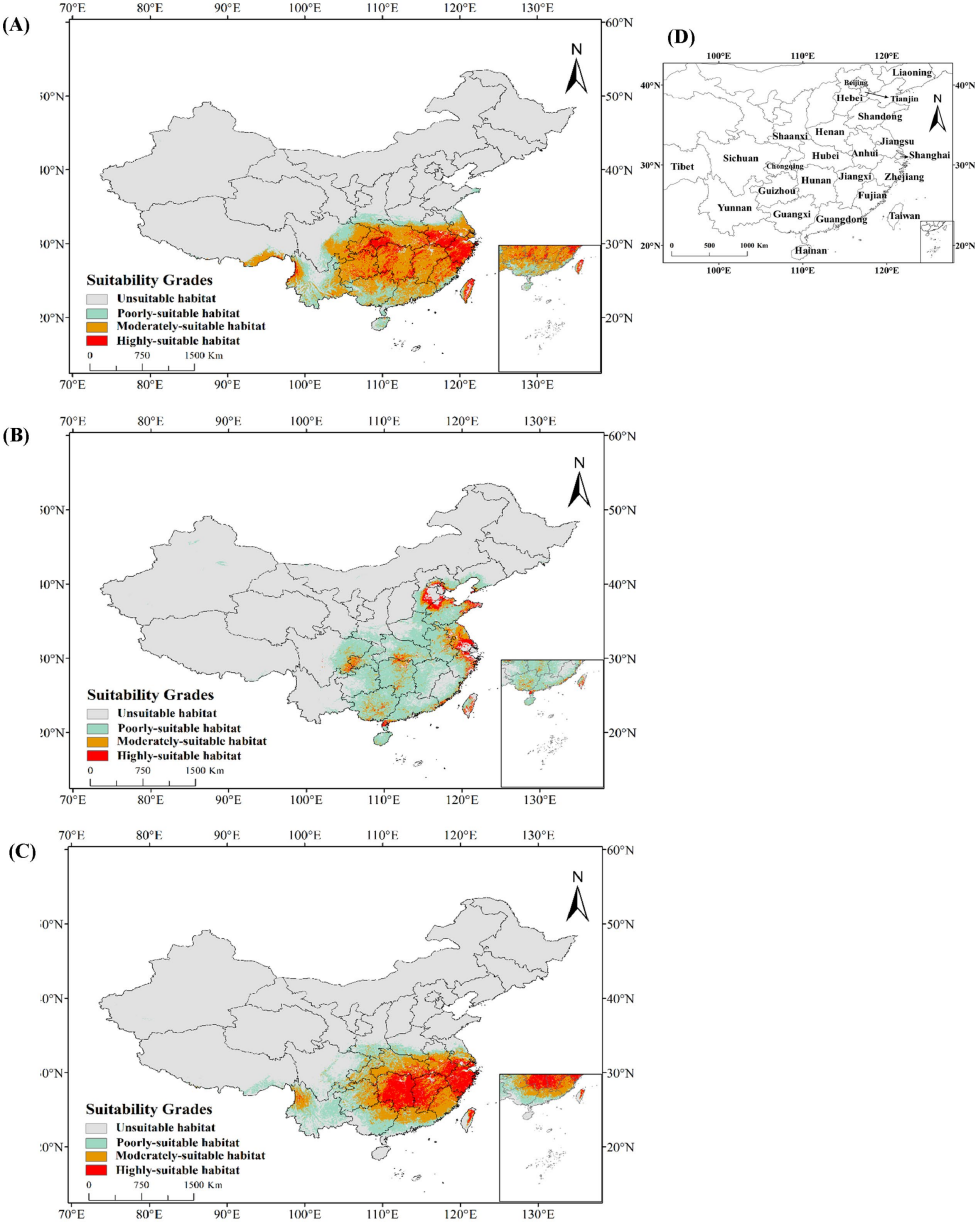
Potential geographical distribution under the current climate in China: **(A)** *C. cicadae*; **(B)** *P. kaempferi*; **(C)** *M. pieli*; **(D)** provinces containing the geographical distributions.

The potential geographic range of *C. cicadae* under present climatic circumstances was mostly concentrated in the southern, central, and eastern areas of China (Figure 7A). This region, covering 214.18 × 10^4^ km^2^, constituted 22.31% of the national land area. The potential distribution range of *M. pieli* closely aligned with that of *C. cicadae* (Figure 7C), while *P. kaempferi*’s potential geographical range extended into the North China region, exhibiting a relative contraction in the southwest (Figure 7B). Suitable habitats for *C. cicadae* were found in the Yunnan Province, eastern Sichuan Province, Guizhou Province, Chongqing City, Hubei Province, Jiangxi Province, Hunan Province, Guangxi Zhuang Autonomous Region, Guangdong Province, central and southern Anhui Province, southern Jiangsu Province, Zhejiang Province, Shanghai City, Taiwan Province and Hainan Province. In contrast, suitable habitats for *P. kaempferi* were concentrated in specific cities on the North China Plain. The highly-suitable areas for *C. cicadae* was 357,900 km^2^, or 3.73% of the national total area. Significant concentrations were identified along the boundary between Guizhou Province and Chongqing City, in the western provinces of Hubei and Hunan, in central and southern Anhui, in Zhejiang Province, and in Taiwan Province. Meanwhile, the highly-suitable areas for *M. pieli* were primarily distributed in cities along the middle and lower reaches of the Yangtze River, covering 44.51 × 10^4^ km^2^, which constituted 4.6% of the national total area. In contrast, the highly-suitable areas for *P. kaempferi we*re mainly located in the Beijing-Tianjin-Hebei region, Weihai City in Shandong Province, Jiangsu Province and Zhejiang Province, covering 11.43 × 10^4^ km^2^, or 1.2% of the national area. Additionally, the area classified as unsuitable habitats for *C. cicadae* encompassed 745.82 × 10^4^ km^2^, representing 86.84% of the national total land area. It was primarily disseminated in the northern, northeastern, and northwestern regions, in addition to the autonomous province of Tibet, and the northeast and northwest regions were also unsuitable for the distribution of both *M. pieli* and *P. kaempferi*. Figure 7D displayed all Chinese provinces with suitable habitats for *C. cicadae* and its two hosts.

#### 3.4.2 Potential geographical distributions under future climate change

**Figure 8:**
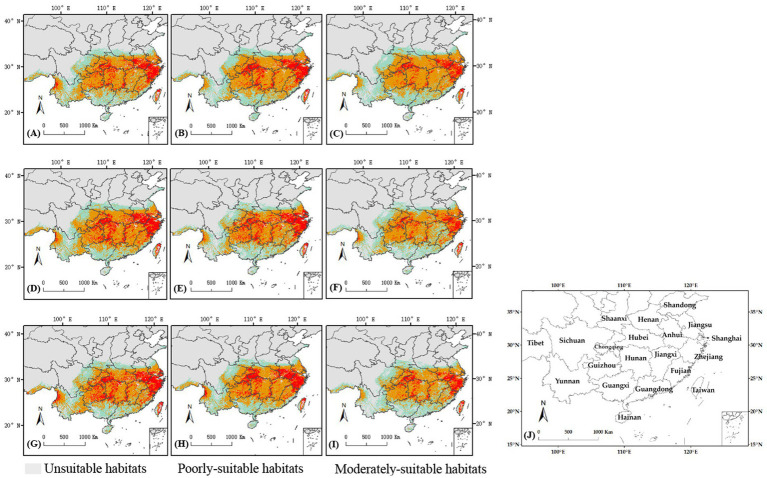
Changes in potential geographical distributions of *C. cicadae* under different climate change scenarios in China: **(A)** SSP126-2030s; **(B)** SSP370-2030s; **(C)** SSP585-2030s; **(D)** SSP126-2050s; **(E)** SSP370-2050s; **(F)** SSP585-2050s; **(G)** SSP126-2070s; **(H)** SSP370-2070s; **(I)** SSP585-2070s; **(J)** provinces containing the geographical distributions.

Figure 8 illustrated the distribution of *C. cicadae* in China under three distinct future temperature scenarios. In the SSP370 scenario, the region classified as unsuitable habitat diminished by 53,100 km^2^ in the 2030s, demonstrating considerable variability. The remaining scenarios, however, remained mostly unaltered. In all three scenarios, the areas of highly-suitable habitat diminished and transitioned into lower suitability classifications. The most significant loss (141,000 km^2^) occurred in the SSP585 scenario.

**Figure 9:**
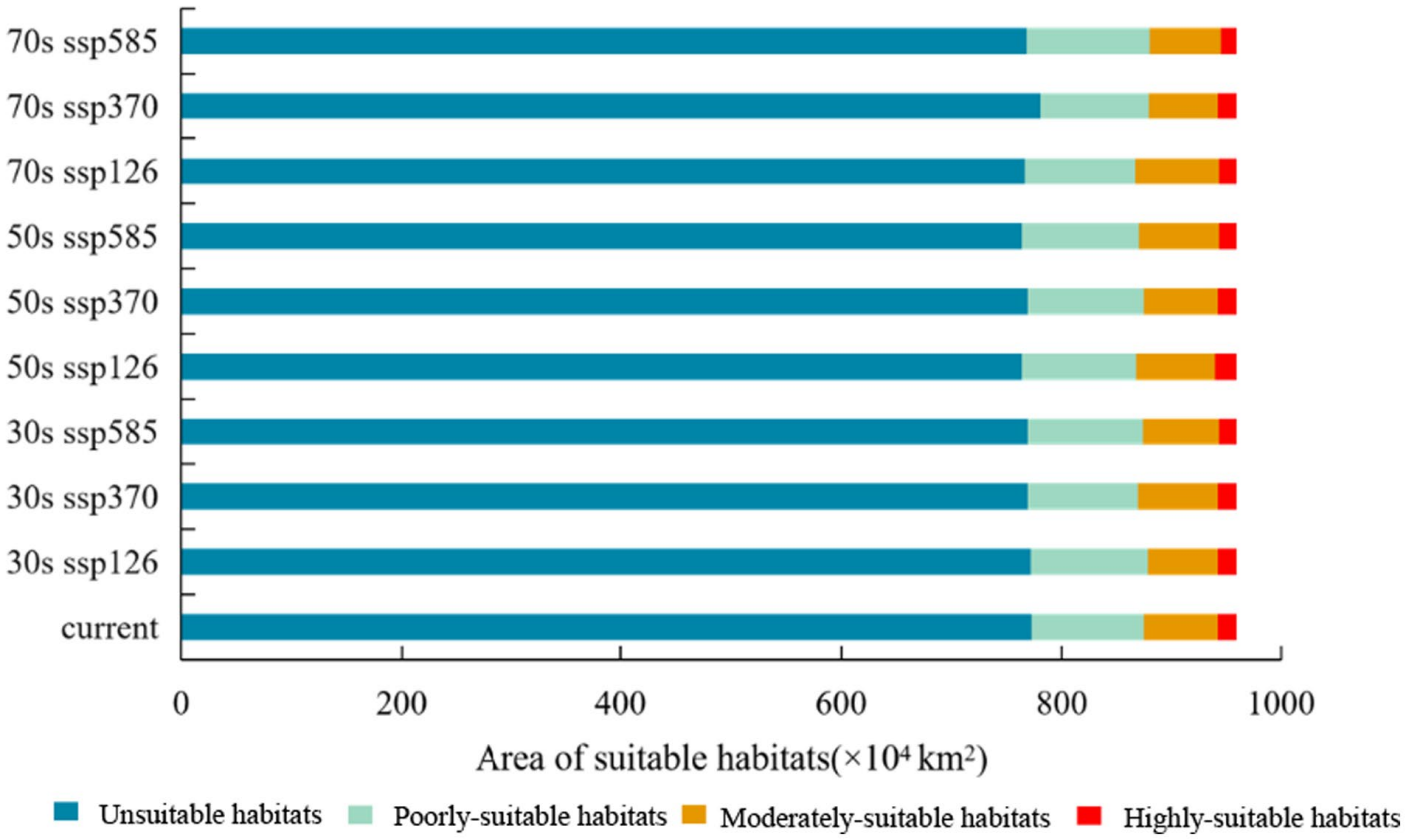
Changed area of suitable habitat between different future climate scenarios and current climate scenario.

The SSP126 scenario indicated a substantial reduction in the area of unsuitable habitat by the 2050s, whereas other scenarios demonstrated rises, with the latter reflecting the most pronounced rise of 210,000 km^2^. Concurrently, the SSP585 scenario’s highly-suitable habitat region (174,000 km^2^) also had the most significant loss. The SSP126 and SSP370 scenarios exhibited varying degrees of increase. Moreover, in every instance, there was an augmentation of unsuitable environments (Figure 9).

Under the SSP370 scenario in the 2050s, the area of *M. pieli*’s unsuitable habitat remained relatively stable, while the highly-suitable habitat area experienced the most significant increase, expanding by 14.3 × 10^4^ km^2^. In contrast, the poorly-suitable habitat area consistently decreased across all scenarios. Notably, the unsuitable habitat area increased under all scenarios, reaching 11.5 × 10^4^ km^2^ and 10.6 × 10^4^ km^2^ in the 2030s under the SSP126 and SSP585 scenarios, respectively, before slightly moderating in the 2050s and 2070s (Supplementary Figure S3).

In all scenarios, the area of unsuitable habitat for *P. kaempferi* increased, with a particularly notable expansion of 9.62 × 10^4^ km^2^ in the SSP126 scenario by the 2050s. Concurrently, the area of highly-suitable habitat decreased across all scenarios. In contrast, the area of poorly-suitable habitat increased in most scenarios (Supplementary Figure S4).

In summary, the SSP126 scenario predominantly exerted a more favorable impact on the distribution of *C. cicadae* across the three time intervals, whereas the SSP585 scenario produced a contrary effect, and the suitable habitat areas exhibited minimal change in the SSP370 scenario, which had the least impact on the distribution of *M. pieli* meanwhile. However, *P. kaempferi*, a main host of *C. cicadae*, its living space in future scenarios were less optimistic.

#### 3.4.3 Analysis of changes in the distribution pattern of habitat

**Figure 10:**
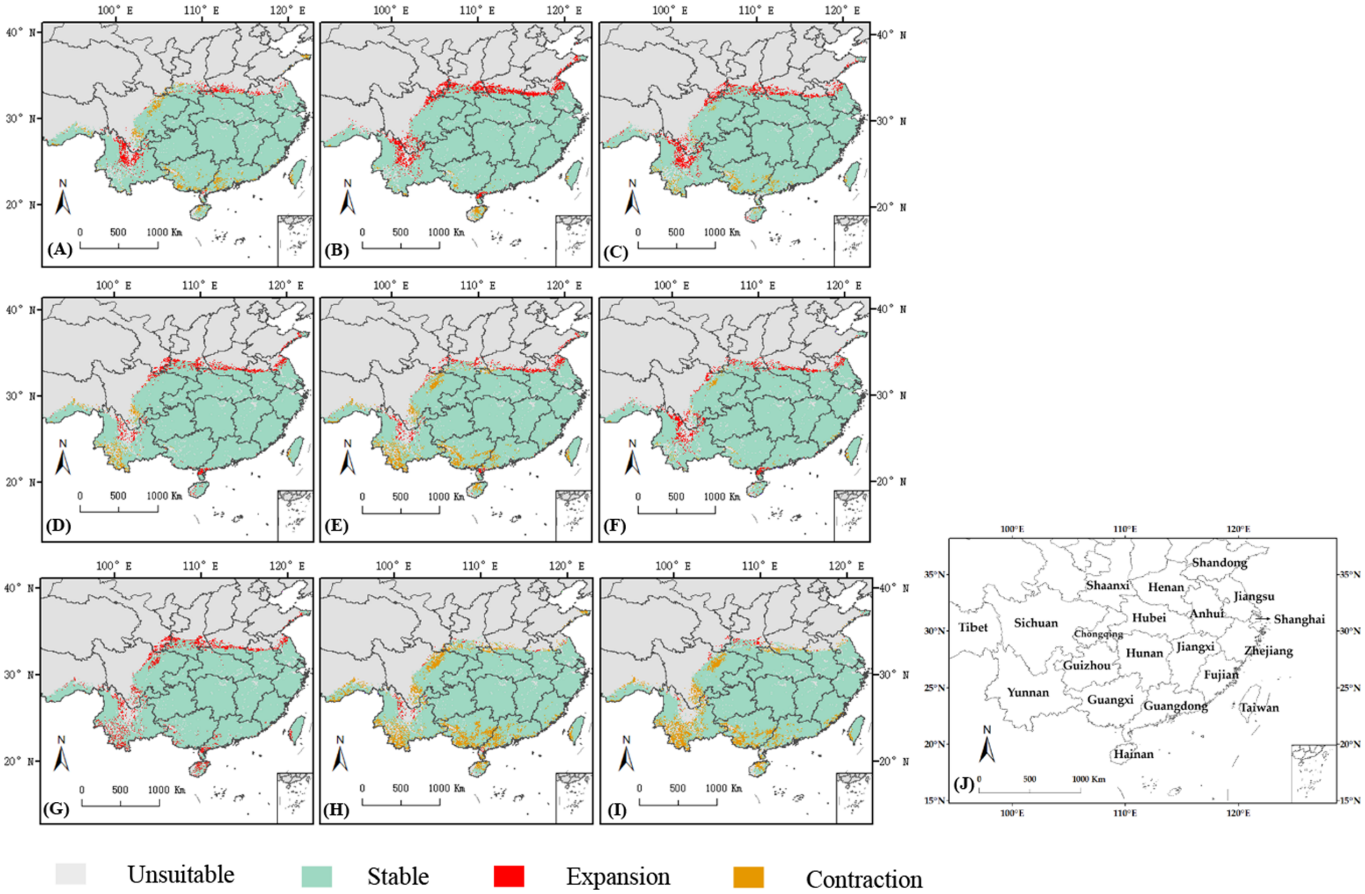
Changes of the suitable habitats of *C. cicadae* between different future climate scenarios and current climate scenario: **(A)** SSP126-2030s; **(B)** SSP126-2050s; **(C)** SSP126-2070s; **(D)** SSP370-2030s; **(E)** SSP370-2050s; **(F)** SSP370-2070s; **(G)** SSP585-2030s; **(H)** SSP585-2050s; **(I)** SSP585-2070s; **(J)** provinces containing the suitable habitats.

The analysis of current and future climatic situations for *C. cicadae* predicted an increase in genetically appropriate habitats under the SSP126 scenario, whereas a reduction was anticipated under the SSP370 and SSP585 scenarios, with the most significant decline occurring in SSP585. Specifically, it was anticipated that in the 2050s and 2070s, suitable habitat areas would diminish by 14.55 km^2^ and 12.34 km^2^, respectively (Table 1). The primary regions exhibiting suitable habitat expansion included northern Yunnan Province, Zhanjiang City in Guangdong Province, and the convergence of the initial suitable areas with the middle and lower reaches of the Yellow River. The regions adjacent to Guangdong Province, southern Guangxi Province, southwestern Yunnan Province, and eastern Sichuan Province were the principal sites of contraction (Figure 10). The expansion and contraction areas of *M. pieli* closely resembled those observed in *C. cicadae* (Supplementary Figure S6), the shared contraction areas were primarily located in the southwestern part of Yunnan Province, the southern part of Sichuan Province, the western part of Guangxi Province, and Jiangmen City in Guangdong Province (Supplementary Figure S7). In contrast, the *P. kaempferi* was sporadically expanding in the Beijing-Tianjin-Hebei region. The areas of contraction were primarily located in central Sichuan and Guizhou, the border between Shaanxi and Sichuan, western Hubei, the border region between Hunan and Guangxi, northern Guangdong, northern Hainan, and most of Jiangxi (Supplementary Figure S5). In addition, the shared contraction zones with the *C. cicadae* were mainly found at the Shaanxi-Sichuan border, northern Hainan, and southwestern Taiwan (Supplementary Figure S7).

**Table 1:** Changes of suitable habitat area of *C. cicadae* under different climate scenarios.

Period	Area (×10^4^ km^2^)		
	Expansion	Contraction	Stable
SSP126-2030s	5.14	5.67	142.21
SSP126-2050s	11.43	1.37	146.51
SSP126-2070s	8.41	3.34	144.55
SSP370-2030s	6.38	2.74	145.14
SSP370-2050s	4.42	8.93	138.95
SSP370-2070s	6.90	1.59	146.29
SSP585-2030s	5.55	5.27	142.61
SSP585-2050s	1.19	15.75	132.13
SSP585-2070s	1.01	13.35	134.53

### 3.5 Changes in the centroids under different climatic scenarios

**Figure 11:**
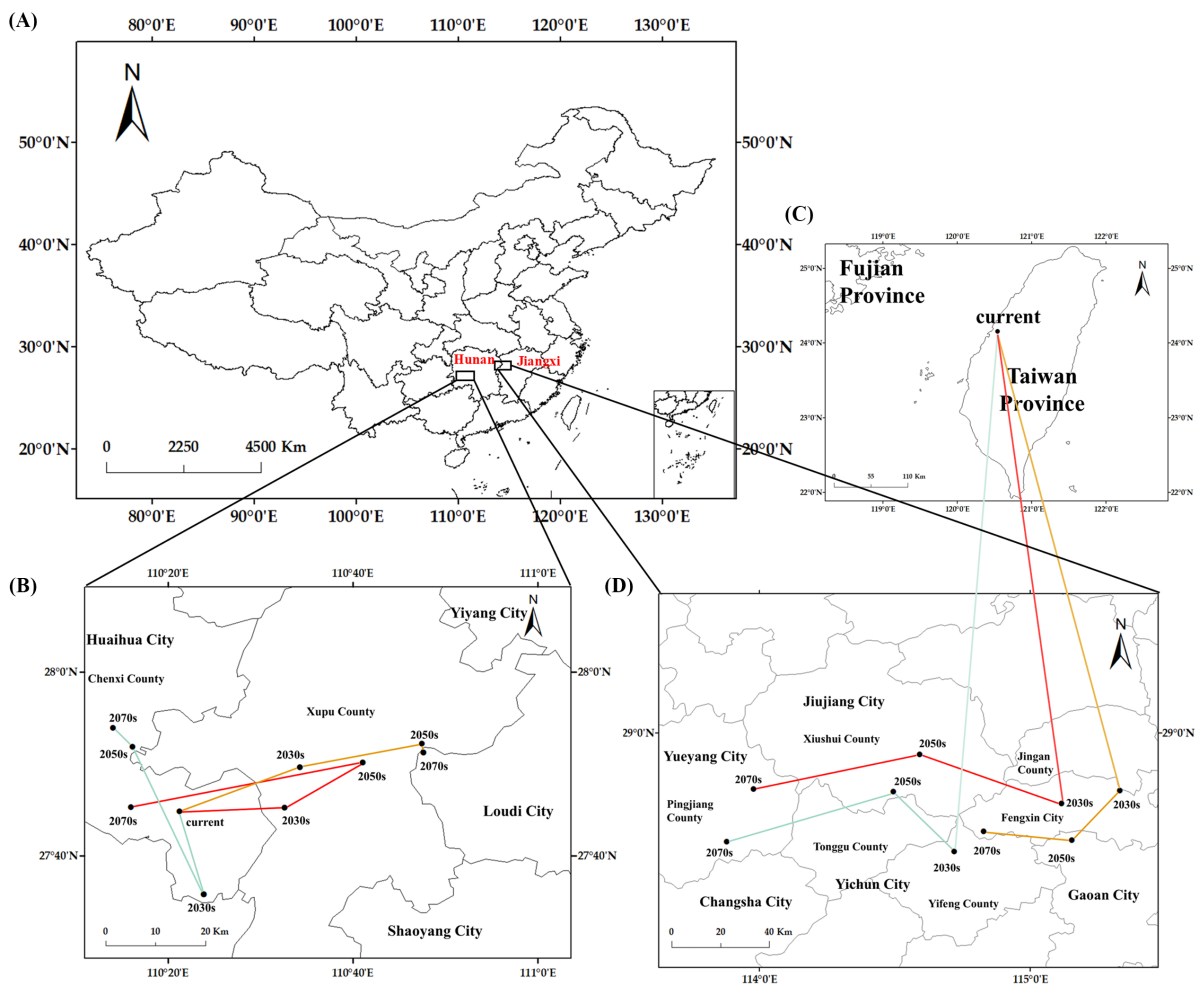
The centroids of suitable habitats under current and future climate scenarios: **(A)** provinces containing the centroids; **(B)** the centroids in the general suitable habitats; **(C)** current centroid in moderately and highly-suitable habitats; **(D)** centroids under future climate scenarios in moderately and highly-suitable habitats.

Hunan Province was identified as possessing both the current and prospective centroids of suitable habitats of *C. cicadae* (Figure 11). The centroid is currently located in Chenxi County, Huaihua City (110°27′E, 27.7459′N). In Chenxi County, all centroids are projected to remain below the SSP126 scenario. The SSP370 and SSP585 scenarios projected an eastward displacement of the centroids. The centroids will be located in Xupu County, Huaihua City, by the 2030s and 2050s. Under the SSP585 scenario by 2070, the centroid will advance eastward to Loudi City (110°47′E, 27°51′N), representing a maximum displacement of 44.97 km from its current position.

The centroids of the moderately and highly appropriate habitats for *C. cicadae* exhibited a broader spectrum of movement. The present centroid was located in Taichung City, Taiwan Province (120°32′E, 24°9′N). Jiangxi and Hunan Provinces were anticipated to be the principal sites of forthcoming centroids. The centroids will transition from Yichun City to Xiushui County in Jiangxi Province in both the SSP126 and SSP370 scenarios. By the 2070s, they will have reached Yueyang City in Hunan Province. The centroid under the SSP585 scenario will be located in Fengxin County and Gao’an City, Jiangxi Province. All future centroids will be situated to the northwest of their present placements.

The current centroid of the total suitable habitat for *M. pieli* was located in Loudi City, Hunan Province (110°27′E, 27°51′N), positioned to the east of the centroid for *C. cicadae*. By the 2070s, this centroid will remain east of the current centroid for *C. cicadae*. Across three future scenarios, the centroid of *M. pieli*’s total suitable habitat showed minimal migration, predominantly remaining in Loudi City, Hunan Province, which coincides with the final position of the centroid for *C. cicadae* under the SSP585 scenario. Additionally, the centroid of *M. pieli*’s medium to high suitability areas primarily migrated within Jiangxi Province, shifting westward by the 2070s compared to the current centroid, aligning with the migration direction of *C. cicadae*’s centroid during the same period. In contrast, the current centroid of the total suitable habitat for *P. kaempferi* was located in Jingzhou City, Hubei Province (113°12′E, 29°56′N). In most scenarios, this centroid tended to migrate northward, a direction inconsistent with the centroid migration of *C. cicadae*. Notably, the centroid of *P. kaempferi*’s medium to high suitability areas exhibited a broad range of movement, spanning three provinces, and was inclined to shift northeastward in the future (Supplementary Figure S8).

Furthermore, in all future scenarios, the elevation of the centroid for *C. cicadae* exceeded that of the current centroid. The centroid of the total suitable habitat for the host *M. pieli* and most centroids of the medium to high suitability areas for *P. kaempferi* also follow this pattern. Interestingly, within the same scenario, the elevation of the centroid for the total suitable habitat of both *C. cicadae* and its host *M. pieli* surpassed that of the centroids for medium and high suitability areas (Supplementary Table S5). However, no significant difference was observed for the host *P. kaempferi*.

## 4 Conclusion

Collectively, predicting climate change is critical for keeping the adequate habitat and diversity of *C. cicadae*. The results indicated that, in the present climate, *C. cicadae* was predominantly located in the southwestern, middle, and eastern parts of China, encompassing 22.31% of the nation’s land area. The highly-suitable habitats were identified in the western provinces of Hubei and Hunan, central and southern Anhui, Zhejiang Province, Taiwan Province, and the border between Guizhou Province and Chongqing City. The potential distribution range of *M. pieli* was similar to that of *C. cicadae*, while *P. kaempferi* extended to the North China Plain. The distribution of *C. cicadae* was predominantly affected by precipitation, followed by temperature, soil, topography and vegetation in secondary roles. The distribution of the host *P. kaempferi*, was closely related to elevation. Future estimates suggested that the SSP126 scenario will enhance the appropriateness for *C. cicadae*, while the SSP585 scenario was detrimental to its dispersion, and the adaptability of host insects especially the *P. kaempferi* will decline in most future scenarios. The northern Yunnan Province and the convergence of the original suitable regions with the middle and lower reaches of the Yellow River were the primary sites of expansion. Conversely, much of the contraction occurred in the eastern region of Sichuan Province, adjacent to southwest Yunnan Province. In the future, centroids for entirely suitable regions will shift to Hunan Province, which encompassed the centroid of the total suitable area for *M. pieli*, but those for moderately and highly-suitable habitats will transition to both Jiangxi and Hunan Provinces, while those for *P. kaempferi* will migrate to Shandong, Jiangsu, and Anhui Provinces. Moreover, in the future, *C. cicadae* and its hosts are likely to migrate to higher elevations, with the elevation of their centroid potentially linked to the suitability of the region where the centroid is located. Our research provided a scientific basis for the conservation and resource management of *C. cicadae*.

## 5 Discussion

### 5.1 Importance of modeling species distributions

The issue of *C. cicadae*’s scarce wild resources has not been sufficiently resolved by the current cultivation methods. The challenge was intensified by the scarcity of research on biomimetic culture tactics and the absence of protocols for cultivating *C. cicadae* in natural environments. The taste and medicinal properties of cultivated *C. cicadae* and those harvested from the wild exhibited notable differences. A link existed between the freshness and sweetness of edible mushrooms and their amino acid concentration (Mau et al., 2001), while functional secondary metabolites exhibited distinct roles (He et al., 2018). Systematic modeling facilitated a thorough comprehension of the influence of various environmental conditions on species distribution and helped predict future trends. This study chose five types of environmental parameters and using the best MaxEnt model to forecast viable habitats for *C. cicadae* under climate change circumstances. The main goals were to determine prevailing environmental parameters and evaluate the effects of future warming scenarios on the distribution and centroid shift of appropriate habitats. Understanding the future distribution of *C. cicadae* can guide proactive strategies for conserving natural resources and alleviating possible threats from climate change. The study’s findings provided significant insights for the efficient management and protection of wild *Cordyceps* resources in China.

### 5.2 Importance of modeling species distributions

Previous studies emphasized the influence of severe cold weather on the distribution of *C. cicadae*, identifying the minimum temperature of the coldest month and the precipitation during the coldest quarter as the primary environmental factors (Zhang et al., 2022). Conversely, our research indicated that the primary environmental element influencing the distribution of *C. cicadae* was the precipitation of the driest month. Both studies recognized the importance of severe monthly environment in shaping distribution patterns. In previous studies, temperature was the predominant variable, however, in the current study, precipitation emerged as the significant factor influencing species distribution modeling. Variations in climate models, species distribution sites, parameter configurations, and the selection of environmental variables may all contribute to this discrepancy. Temperature and precipitation were the principal contributors to the model, even after incorporating three more environmental factors into our analysis. Field experiments in Sichuan Province verified these results, demonstrating a significant correlation between temperature, relative humidity, and the growth density of *C. cicadae* (Zhang et al., 2022). The temperature trend in our study corresponded with the annual average temperature, demonstrating that growth density initially increased with rising temperatures before subsequently falling. A comparable trend associated with precipitation was observed in relative humidity, illustrating the indisputable influence of climate. Furthermore, low temperatures and relative humidity impeded the proliferation of insect pathogenic fungi, hence restricting their ability to infect hosts, as demonstrated by trials simulating insect infections (Boaventura et al., 2021). We suggested that a mechanism existed through which fluctuations in temperature and precipitation influenced *Cordyceps* distribution. Consequently, we proposed two strategies: Initially, enhance research on forest-floor bionic cultivation techniques; subsequently, initiate breeding programs for *C. cicadae* to augment resistance to adverse conditions, aiming to develop drought-tolerant, cold-resistant, and heat-resistant varieties; finally, adjust the species composition of *Cordyceps* according to climate zoning and establish cultivation bases for *C. cicadae* in suitable regions, akin to other edible fungi.

This study corroborated Ruan’s findings that the distribution of *C. cicadae* was significantly influenced by soil pH (Huang et al., 2021). Ruan’s research indicated that a soil pH of 5.9 was optimal for *C. cicadae*, aligning with the suitable pH range identified in this study. Notably, the pH of soils containing *C. cicadae* was significantly lower than that of soils devoid of them. This study observed similar pH change patterns in both the *C. cicadae* and its host insect, *M. pieli*. The transfer of material between the soil and *C. cicadae* is one reason for this phenomenon. The host insects of *C. cicadae* reside in the soil for extended periods during their larval stage. The endophytic fungus of *C. cicadae* assimilated carbon and nitrogen from the soil and converted them into its own carbon-nitrogen compounds (Qu et al., 2019). It can also absorb soil pollutants such as organophosphate esters (OPEs) and polycyclic aromatic hydrocarbons (PAHs) (Shimazu, 2015). These elements influenced the transport and conversion mechanisms that facilitated the growth and development of *C. cicadae*. Therefore, in areas of low appropriateness, it is essential to implement localized techniques to reduce soil pH. It is recommended to implement certain measures, including the application of humus soil, the cultivation of green manure crops, and the modification of leaching irrigation, while considering the growing conditions of the surrounding vegetation.

The slope is a topographic feature that influences the vertical properties of soil and indirectly affects species distribution (Kebede and Negassa, 2023). Research indicated that an increase in slope correlated with a decrease in soil pH. This study found that suitability improved as soil pH decreased, which aligned with the result that steeper slopes also enhanced suitability. Surveys done in Sichuan Province, however, revealed an opposing trend: suitability initially increased and then decreased with slope. The gap may be attributed to the Sichuan Basin’s subtropical monsoon climate, characterized by abundant rainfall. The steep slopes of this location heightened the risk of species extinction and soil erosion. To mitigate soil erosion, the cultivation of deep-rooted vegetation and regular monitoring in steep areas should be promoted.

### 5.3 Analysis of future changes in distribution and improvements

This study utilized three climate models: EC-Earth3-Veg, MPI-ESM1-2-HR, and BCC-CSM2-MR. In Asia, these General Circulation Models (GCMs) were frequently utilized in climate change research. The precision with which BCC-CSM2-MR simulated China’s actual climate conditions was particularly remarkable. Research employing observational constraints demonstrated that this model accurately depicted China’s temperature and precipitation distribution (Liu et al., 2022; Ju et al., 2023). In the Southeast, it tended to under-estimate summer precipitation. In contrast, the EC-Earth3-Veg model excelled at predicting rainfall patterns in Asia (Pimonsree et al., 2023). The MPI-ESM1-2-HR model excelled in simulating the frequency of Pacific Blocking (PBF) occurrences associated with severe winter weather (Gao et al., 2022). It was capable of adjusting to fluctuations in forthcoming episodes of severe cold. Moreover, MPI-ESM1-2-HR exhibited a significant ability to predict drought conditions by accurately assessing the incidence of drought events (Yang et al., 2023). Integrating these three climate models can mitigate uncertainties that may arise from relying on a single model.

Huang’s research indicated that a future climatic scenario will substantially reduce the region suited for *C. cicadae*’s habitat (Zhang et al., 2022). This pattern corresponded with our findings for the SSP585 scenario. Nonetheless, SSP126 and SSP370 were additional climate scenarios included in our research. The SSP370 scenario indicated a constant effect on the suitable habitat area, while the SSP126 scenario exhibited a somewhat favorable influence. Conversely, the SSP585 scenario resulted in a notable reduction in the medium to high suitability habitats. The suitability of habitats for *C. cicadae* was significantly affected by elevated greenhouse gas emissions and fossil fuel usage. Mitigating carbon emissions and replacing fossil fuels with renewable energy sources, such as biomass, is crucial for alleviating these impacts. Moreover, altering vegetation types and incorporating local species can enhance forests’ capacity to store carbon, purify the air, and alleviate the adverse impacts of climate change.

Further analysis indicated that, in the SSP585 scenario, drought stress was increasing in several regions of China. For instance, a projected intensity of heat stress was anticipated across the North China Plain (Xue et al., 2024). In the provinces of Anhui and Jiangsu, areas currently considered moderately to highly suitable for habitation, may experience direct impacts on their ecosystems. Similarly, it was expected that the drought in tropical regions, including Hainan Province, southwest Yunnan Province, and southern Taiwan, would intensify (Nauditt et al., 2022; Tian et al., 2023). In particular, our study’s predictions for future habitat suitability supported the hypothesis that southwest Yunnan and Hainan, which are currently categorized as poorly-suitable habitats, could eventually become unsuitable areas. This highlighted that the decrease in appropriate habitat areas was primarily due to drought.

Moreover, it was concerning that the availability of suitable habitats for *C. cicadae* and its host, *M. pieli* in the Sichuan Basin and Lingnan region would significantly diminish under most future climatic scenarios. The *P. kaempferi* had shown a noticeable decline in Jiangxi, Fujian, and cities along the middle and lower reaches of the Yangtze River. Consequently, to ensure the resilience of *C. cicadae* growth habitats to climate change, it is crucial to formulate customized conservation strategies. The design of rainfall gathering and forest self-irrigation systems are vital for optimizing water resource utilization in drought-prone regions. Minimizing soil moisture evaporation can be accomplished by implementing measures such as artificially aerating the soil in forested areas. It is imperative to augment the quantity of parks and nature reserves in these areas and to implement explicit regulations constraining growth. Establishing a long-term monitoring system and safeguarding *C. cicadae* germplasm resources in suboptimal conditions is also essential.

Regardless of varying pathways, the centroid of appropriate habitats tended to shift to higher elevations under future scenarios. The migration of host insects, compelled by climate change to ascend to higher elevations, was a significant contributing factor. It is essential to acknowledge that insects with inferior migration and adaptation capabilities will be eradicated, leading to a decrease in habitable space or potential extinction, despite the precise causes remaining uncertain. By 2050, 15–37% of the 1,100 insect species may face extinction due to climate change (Raza et al., 2015). In this study, the suitable habitat for host insects, particularly *P. kaempferi*, was projected to significantly shrink under most future scenarios. Therefore, alongside the preservation of *C. cicadae*, it was imperative to safeguard the genetic resources of *P. kaempferi* and *M. pieli*. Establishment of a germplasm bank for host insects was recommended.

### 5.4 Research limitations

This study emphasized the significance of protecting appropriate habitats and species diversity to counteract the risks that global warming poses to the natural resources of *C. cicadae*. However, there were still discrepancies between predictions and reality because field surveys and observational data were lacking. Future projects should prioritize doing field surveys and gathering data in the indicated regions.

## Data Availability

The original contributions presented in this study are included in the article and Supplementary material. For further inquiries, please contact the corresponding author.

## Footnotes

^1^http://www.gbif.org; accessed on May 28, 2024.

^2^https://www.cfh.ac.cn; accessed on May 28, 2024.

^3^https://nmdc.cn/fungarium/fungi/chinastrain; accessed on May 28, 2024.

^4^http://nsii.org.cn/2017/home.php; accessed on May 28, 2024.

^5^https://portal.taibif.tw/zh-hant/; accessed on May 28, 2024.

^6^ https://www.ovital.com/

^7^https://data.tpdc.ac.cn/home; accessed on 8 June 2024.

^8^https://www.gscloud.cn/; accessed on 8 June 2024.

^9^https://www.resdc.cn/; accessed on 8 June 2024.

^10^http://worldclim.org; retrieved on 8 June 2024.

^11^ http://www.sdmtoolbox.org/downloads
