# Supplementary material for: Potential geographical distribution of Cordyceps cicadae and its two hosts in China under climate change
Source: Front Microbiol. 2025 Jan 15;15:1519560. doi: 10.3389/fmicb.2024.1519560 (PMC11778177; doi:10.3389/fmicb.2024.1519560)
Supplement: Supplementary file 1 [file Data_Sheet_1.docx]

Supplementary Material

**Table S1 28 environmental variables used in this Study**

| Name | Variables and description | Unit |
| --- | --- | --- |
| bio1 | annual mean temperature | ℃ |
| bio2 | mean diurnal range(mean of monthly (max temp - min temp) | ℃ |
| bio3 | isothermality (bio2/bio7) (* 100) | / |
| bio4 | temperature seasonality (standard deviation *100) | / |
| bio5 | max temperature of warmest month | ℃ |
| bio6 | min temperature of coldest month | ℃ |
| bio7 | temperature annual range (bio5-bio6) | ℃ |
| bio8 | mean temperature of wettest quarter | ℃ |
| bio9 | mean temperature of driest quarter | ℃ |
| bio10 | mean temperature of warmest quarter | ℃ |
| bio11 | mean temperature of coldest quarter | ℃ |
| bio12 | annual precipitation | mm |
| bio13 | precipitation of wettest month | mm |
| bio14 | precipitation of driest month | mm |
| bio15 | precipitation seasonality (coefficient of variation) | / |
| bio16 | precipitation of wettest quarter | mm |
| bio17 | precipitation of driest quarter | mm |
| bio18 | precipitation of warmest quarter | mm |
| bio19 | precipitation of coldest quarter | mm |
| pH | soil pH(H_2_O) value | / |
| SOM | soil organic matter | % |
| TP | total phosphorus | % |
| TK | total potassium | % |
| AP | available phosphorus | ppm |
| AK | available potassium | ppm |
| elevation | elevation | m |
| slope | slope | % |
| vege | vegetation type | / |

**Table S2 Contribution of each environmental variables to the distribution of *C. cicadae*.**

| **Name** | **Description** | **Percent**  **Contribution/%** | **Permutation**  **Importance/%** |
| --- | --- | --- | --- |
| bio14 | precipitation of driest month(mm) | 54.2 | 5.6 |
| bio12 | annual precipitation(mm) | 8.4 | 0.9 |
| bio1 | annual mean temperature(℃) | 6.5 | 3.4 |
| slope | slope(%) | 2.5 | 2.1 |
| bio3 | isothermality (bio2/bio7) (* 100) | 2.1 | 1 |
| bio15 | precipitation seasonality (coefficient of variation) | 1.6 | 3.8 |
| bio18 | precipitation of warmest quarter(mm) | 1.4 | 10.9 |
| pH | soil pH(H_2_O) value | 1.4 | 1.1 |
| bio10 | mean temperature of warmest quarter(℃) | 1.3 | 2.3 |
| bio2 | mean diurnal range(mean of monthly (max temp - min temp)(℃) | 1.1 | 2 |
| AP | available phosphorus(ppm) | 1 | 0.7 |
| vege | vegetation type | 1 | 1 |
| TP | total phosphorus(%) | 0.7 | 0.6 |
| AK | available potassium(ppm) | 0.7 | 0.9 |

**Table S3 Contribution of each environmental variables to the distribution of** ***P. kaempferi*.**

| **Name** | **Description** | **Percent**  **Contribution/%** | **Permutation**  **Importance/%** |
| --- | --- | --- | --- |
| elevation | elevation(m) | 18.8 | 1.5 |
| bio14 | precipitation of driest month(mm) | 11.7 | 0.4 |
| bio16 | precipitation of wettest quarter(mm) | 10.7 | 3.2 |
| bio6 | min temperature of coldest month(℃) | 10.3 | 24.1 |
| bio15 | precipitation seasonality (coefficient of variation) | 10.3 | 6 |
| bio2 | mean diurnal range(mean of monthly (max temp - min temp)(℃) | 9.6 | 2.8 |
| vege | vegetation type | 3.6 | 0.7 |
| bio3 | isothermality (bio2/bio7) (* 100) | 2.9 | 2 |
| bio8 | mean temperature of wettest quarter(℃) | 1.9 | 22.2 |
| TP | total phosphorus(%) | 1.4 | 1 |
| slope | slope(%) | 1.2 | 2 |
| SOM | soil organic matter(%) | 1 | 2.2 |
| AP | available phosphorus(ppm) | 1 | 1.5 |
| pH | soil pH(H_2_O) value | 0.7 | 0.3 |

**Table S4 Contribution of each environmental variables to the distribution of *M. pieli*.**

| **Name** | **Description** | **Percent**  **Contribution/%** | **Permutation**  **Importance/%** |
| --- | --- | --- | --- |
| bio14 | precipitation of driest month(mm) | 34.7 | 0.8 |
| AP | available phosphorus(ppm) | 3.2 | 2.5 |
| bio6 | min temperature of coldest month(℃) | 3 | 18.9 |
| SOM | soil organic matter(%) | 3 | 5.4 |
| bio15 | precipitation seasonality (coefficient of variation) | 2.8 | 2.6 |
| slope | slope(%) | 2.4 | 2 |
| bio9 | mean temperature of driest quarter(℃) | 1.5 | 6.3 |
| pH | soil pH(H_2_O) value | 1.5 | 2.2 |
| vege | vegetation type | 1.5 | 0.6 |
| bio10 | mean temperature of warmest quarter(℃) | 1.3 | 3.7 |
| AK | available potassium(ppm) | 1.2 | 0.7 |
| bio2 | mean diurnal range(mean of monthly (max temp - min temp)(℃) | 0.7 | 3.1 |
| bio16 | precipitation of wettest quarter(mm) | 0.7 | 2.1 |
| TP | total phosphorus(%) | 0.6 | 0.6 |

**
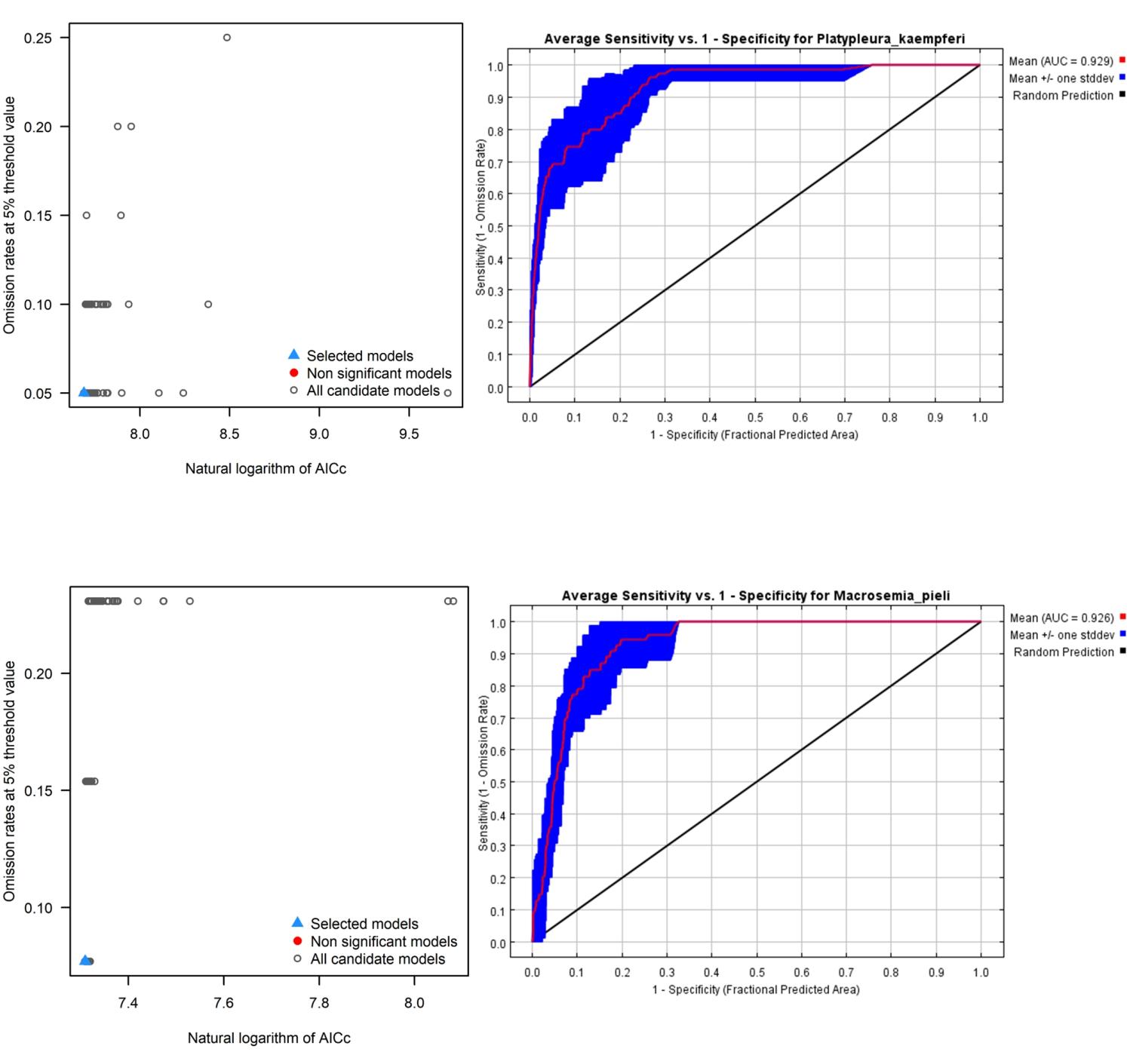
**

**(B)**

**(A)**

**(D)**

**(C)**

**FIGURE S1 (A-D)** MaxEnt model parameter calibration results: (A) Results of the selection of the optimal models for *M. pieli*; (B) ROC curves and AUC values of MaxEnt model prediction results for *M. pieli*; (C) Results of the selection of the optimal models for *P. kaempferi*; (D) ROC curves and AUC values of MaxEnt model prediction results for *P. kaempferi*. The black line represented random prediction, whereas the red curve represented training data.


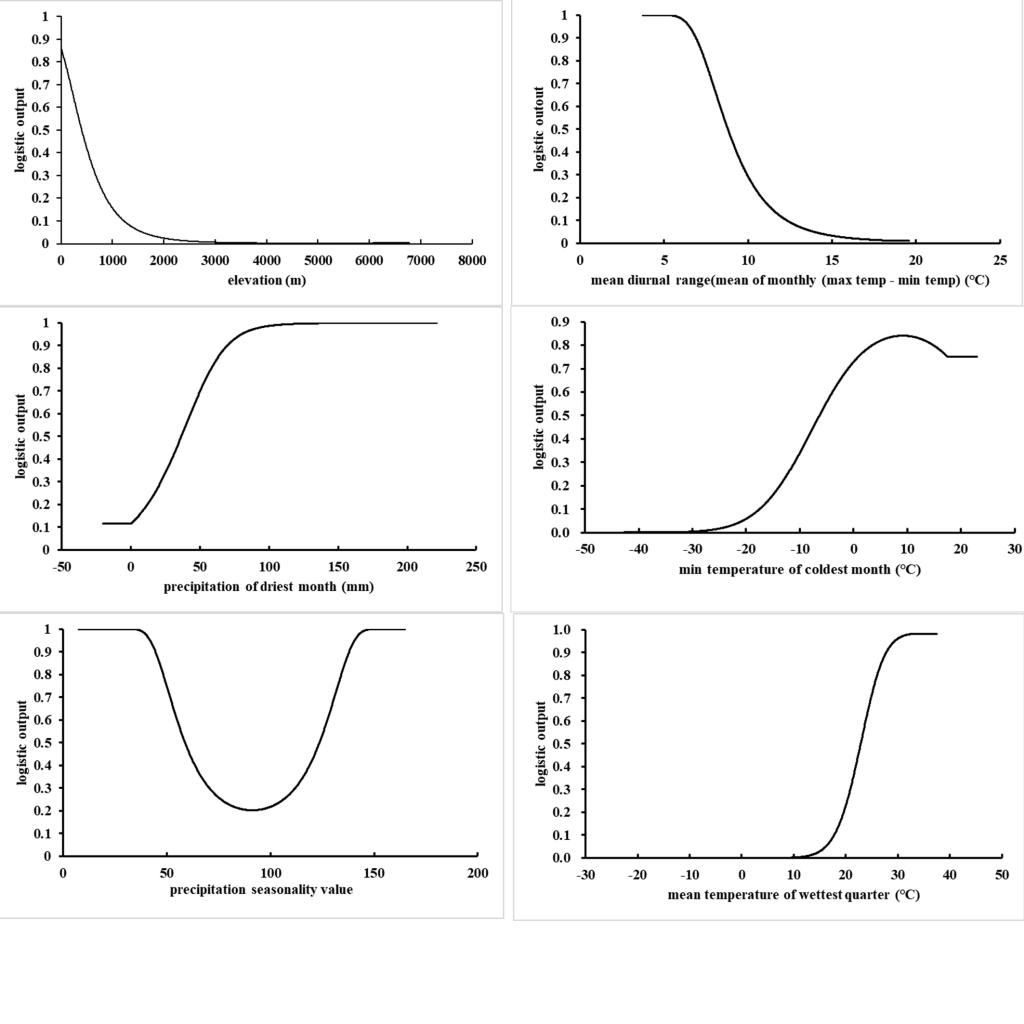


**(A)**

**(B)**

**(C)**

**(D)**

**(E)**

**(F)**

**
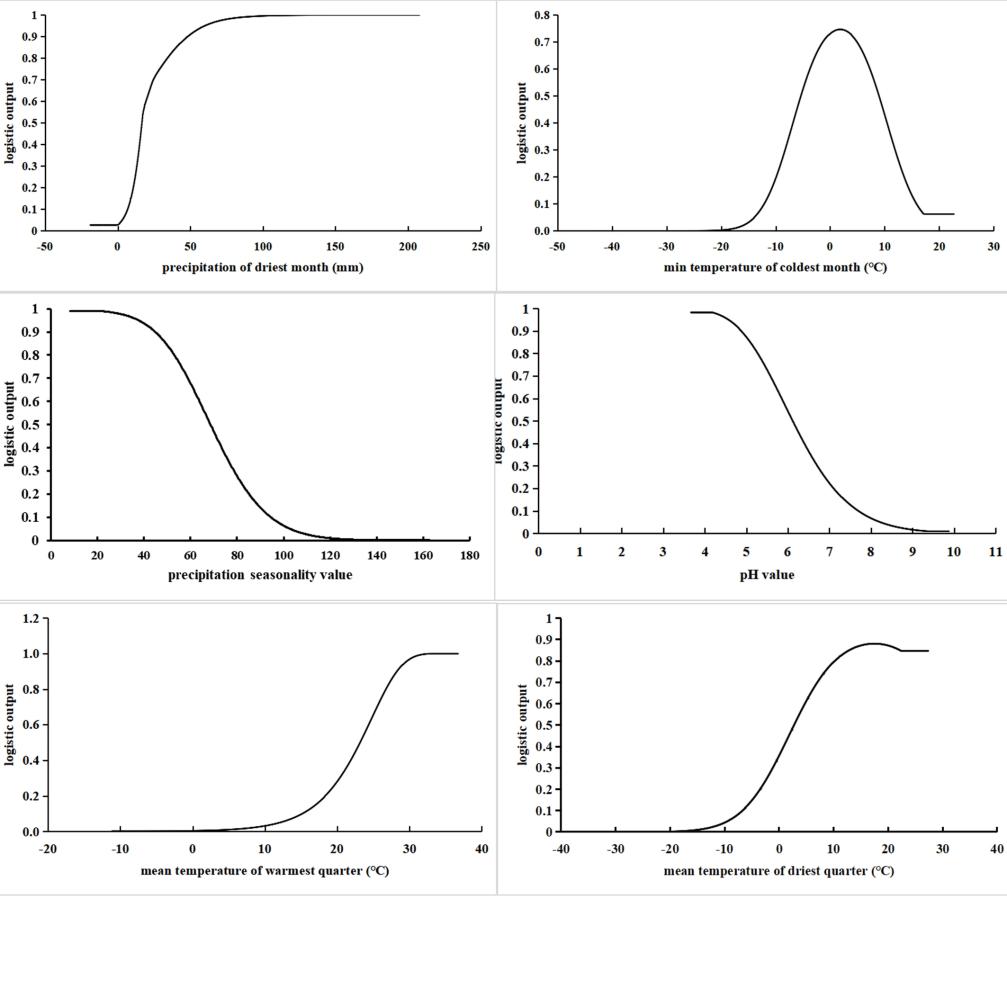
**

**(a)**

**(b)**

**(c)**

**(d)**

**(e)**

**(f)**

**FIGURE S2 (A-F)** Response curves of the probability of *P. kaempferi* presence to dominant environmental variables: (A) elevation; (B) bio2; (C) bio14; (D) bio6; (E) bio15; (F) bio8. **(a-f)** Response curves of the probability of *M. pieli* presence to dominant environmental variables: (a) bio14; (b) bio6; (c) bio15; (d) pH; (e) bio10; (f) bio8.


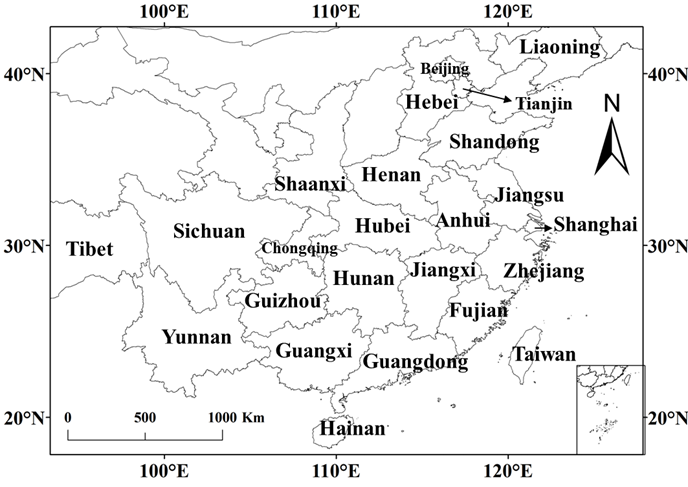


**(j)**


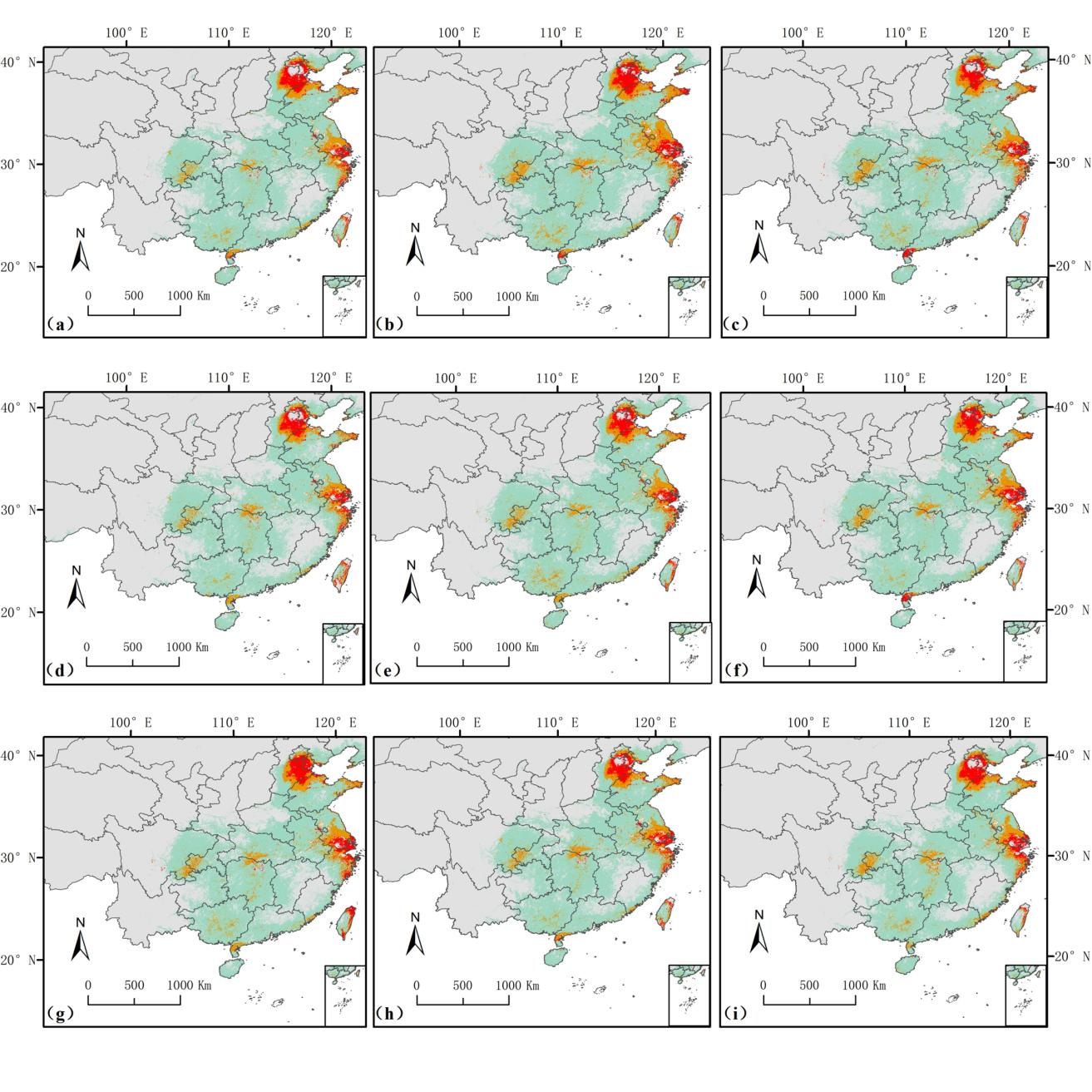


Highly-suitable habitats

Moderately-suitable habitats

Poorly-suitable habitats

Unsuitable habitats

**FIGURE S3 (a-j)** Changes in potential geographical distributions of *P. kaempferi* under different climate change scenarios in China: (a) SSP126-2030s; (b) SSP370-2030s; (c) SSP585-2030s; (d) SSP126-2050s; (e) SSP370-2050s; (f)SSP585-2050s; (g) SSP126-2070s; (h) SSP370-2070s; (i) SSP585-2070s; (j) provinces containing the geographical distributions.


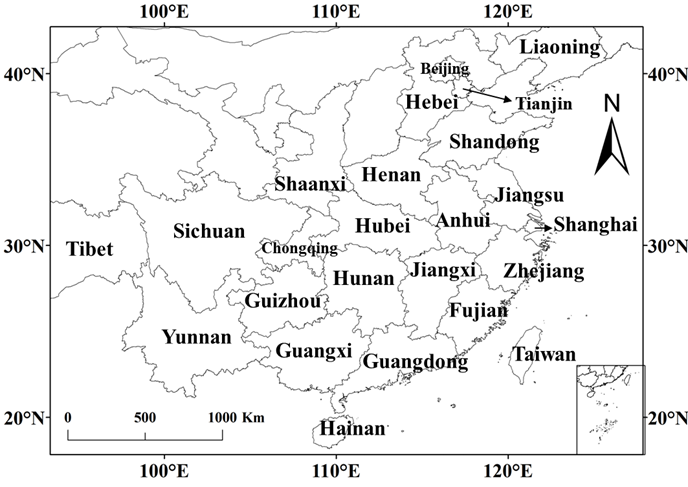


**(j)**


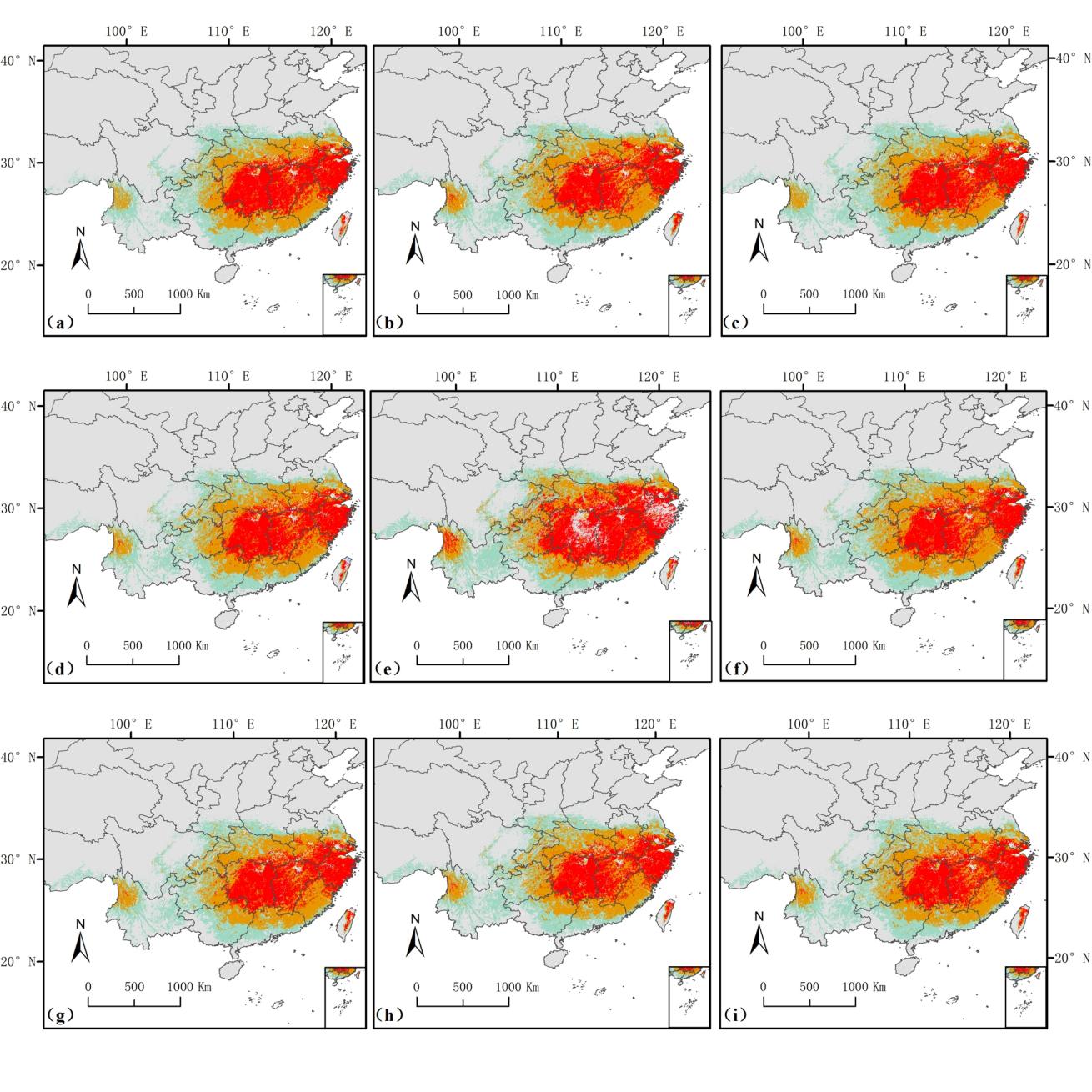


Highly-suitable habitats

Moderately-suitable habitats

Poorly-suitable habitats

Unsuitable habitats

**FIGURE S4 (a-j)** Changes in potential geographical distributions of *M. pieli* under different climate change scenarios in China: (a) SSP126-2030s; (b) SSP370-2030s; (c) SSP585-2030s; (d) SSP126-2050s; (e) SSP370-2050s; (f)SSP585-2050s; (g) SSP126-2070s; (h) SSP370-2070s; (i) SSP585-2070s; (j) provinces containing the geographical distributions.


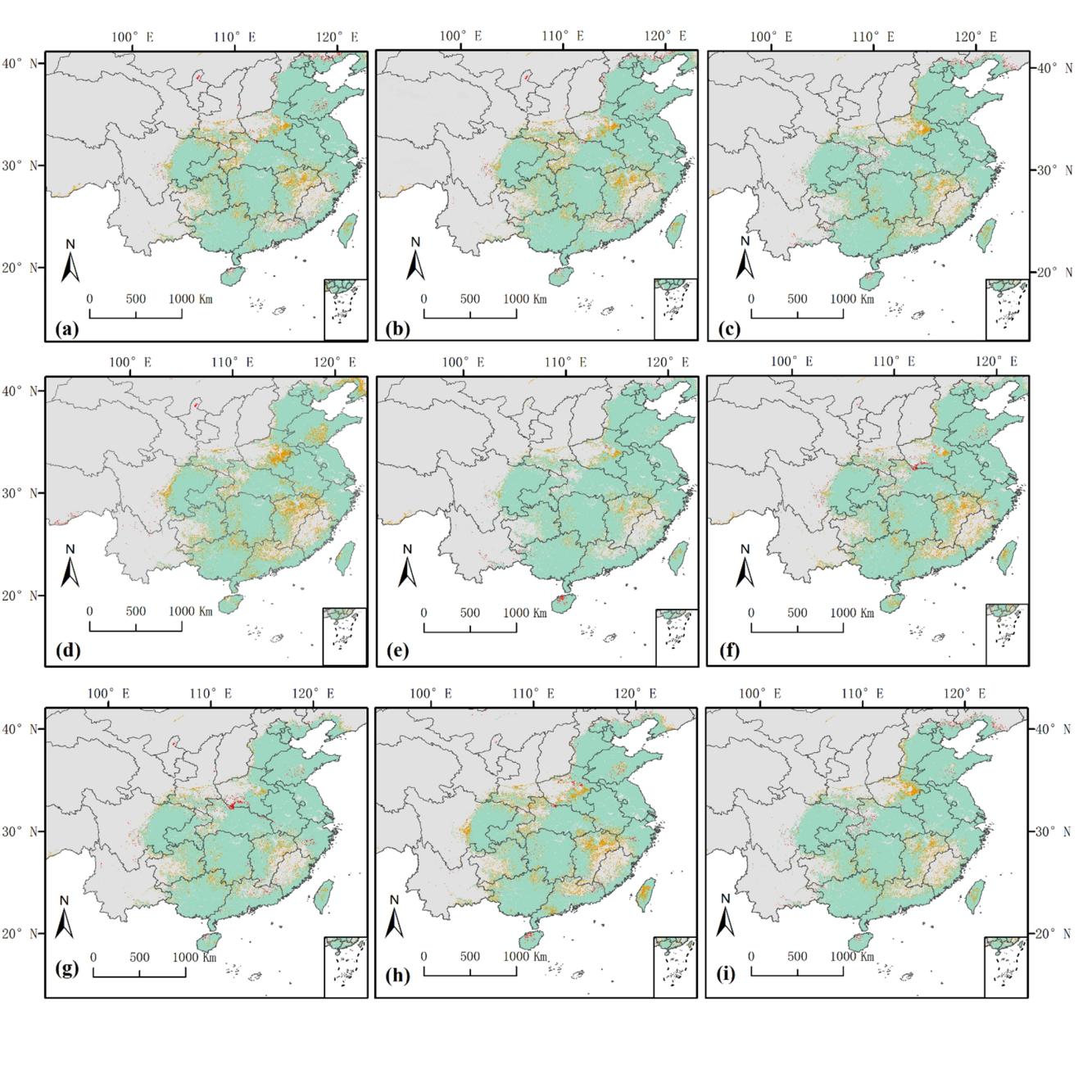

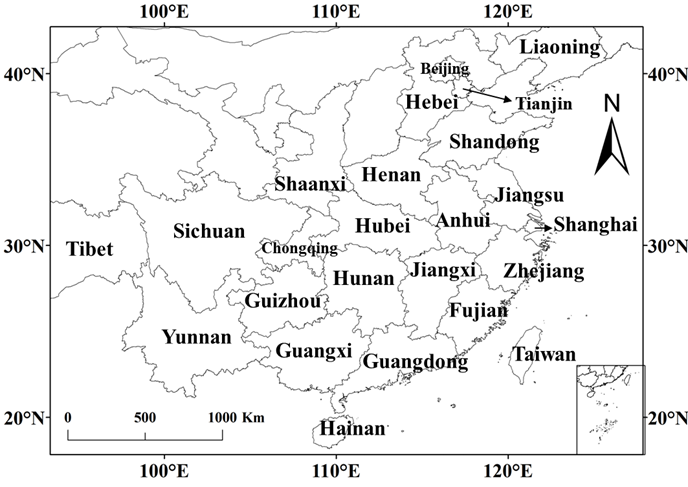


**(j)**

Unsuitable

Expansion

Contraction

Stable

**FIGURE S5 (a-j)** changes of the suitable habitats of P. kaempferi between different future climate scenarios and current climate scenario: (a) SSP126-2030s; (b) SSP370-2030s; (c) SSP585-2030s; (d) SSP126-2050s; (e) SSP370-2050s; (f) SSP585-2050s; (g) SSP126-2070s; (h) SSP370-2070s; (i) SSP585-2070s; (j) provinces containing the suitable habitats.


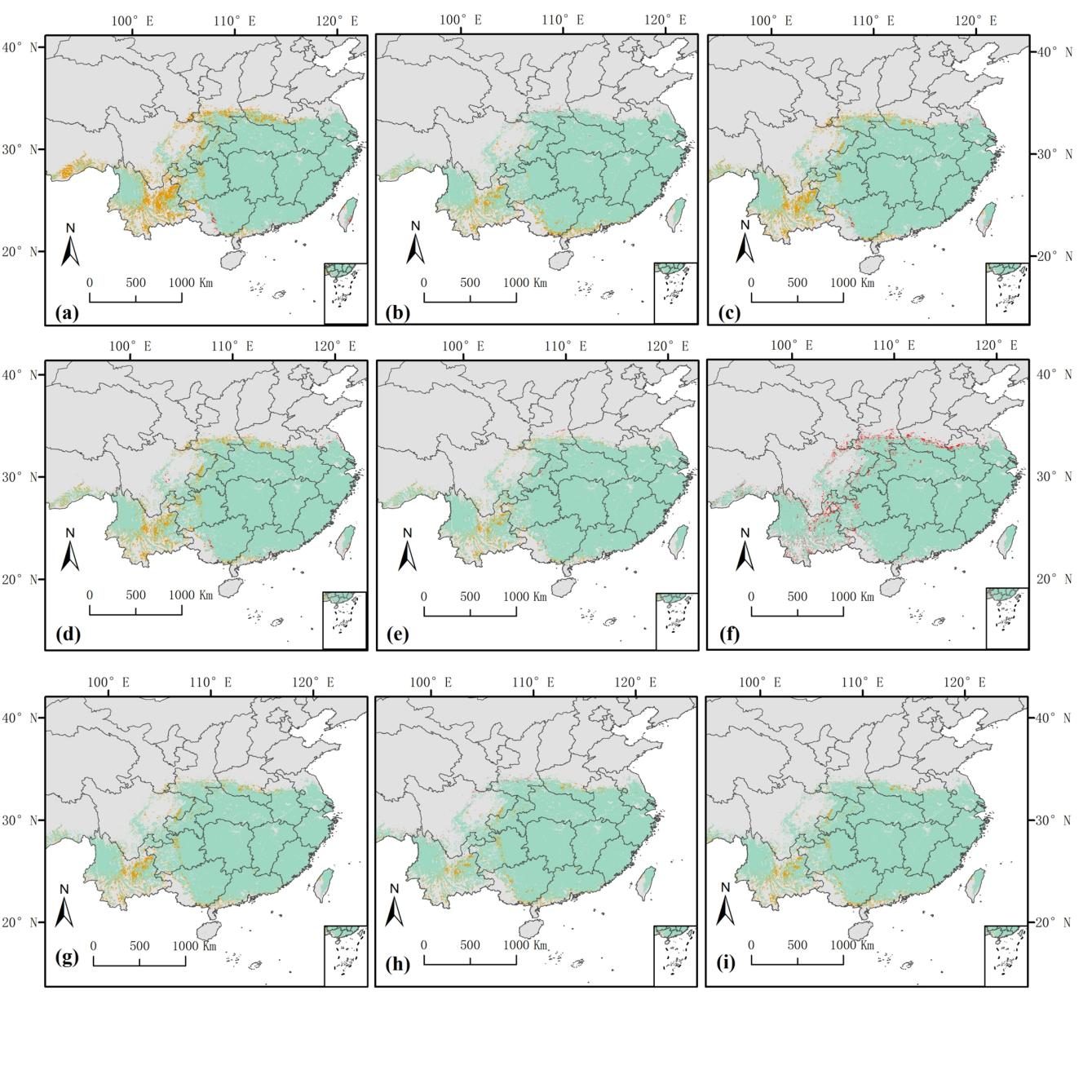

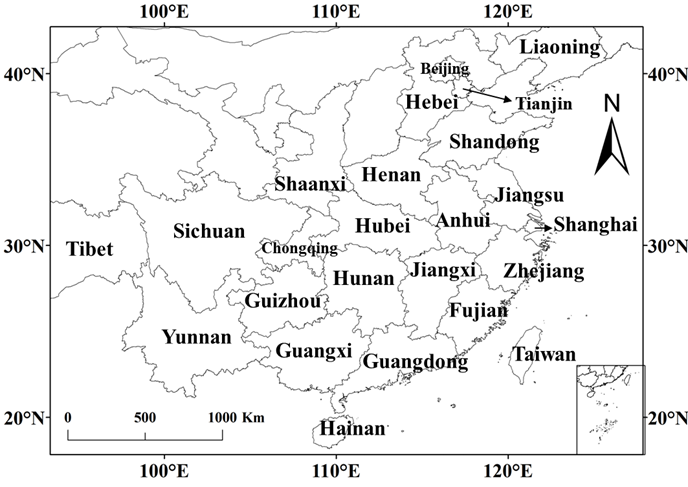


**(j)**

**(j)**

Unsuitable

Expansion

Contraction

Stable

**FIGURE S6 (a-j)** changes of the suitable habitats of *M. pieli* between different future climate scenarios and current climate scenario: (a) SSP126-2030s; (b) SSP370-2030s; (c) SSP585-2030s; (d) SSP126-2050s; (e) SSP370-2050s; (f) SSP585-2050s; (g) SSP126-2070s; (h) SSP370-2070s; (i) SSP585-2070s; (j) provinces containing the suitable habitats.


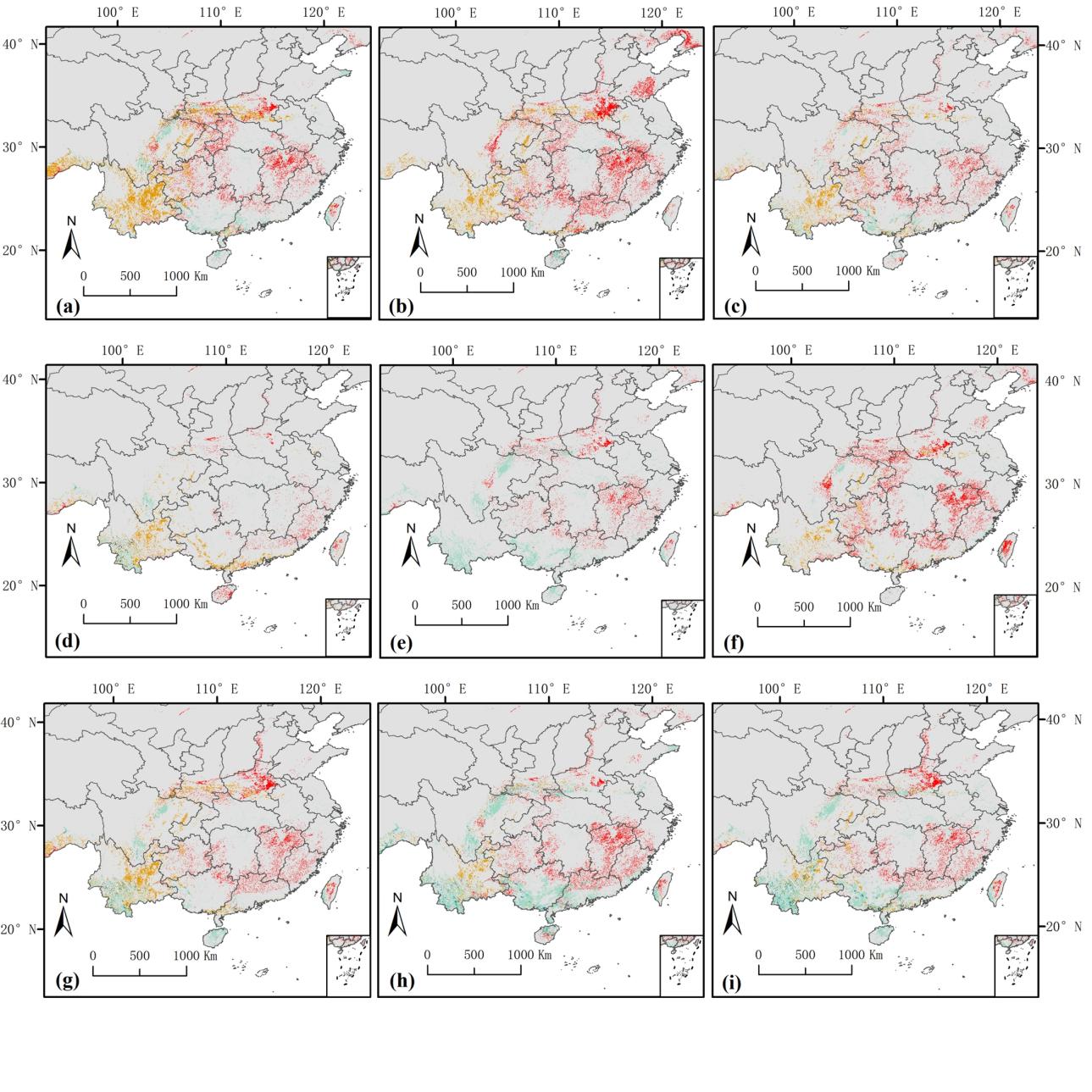

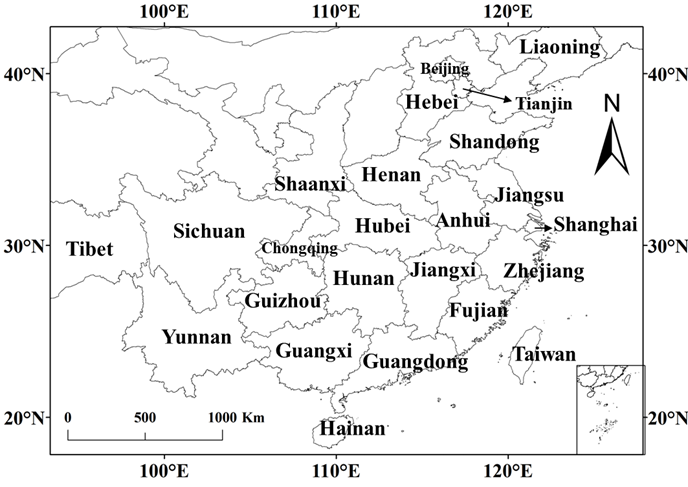


**(j)**

Contraction region(P and M)

Contraction region(M)

Contraction region(P)

Non-contraction region(P, M and C)

Contraction region(M and C)

Contraction region(P, M and C)

Contraction region(P and C)

Contraction region(C)

**FIGURE S7 (a-j)** the analysis of the overlap in the contraction areas of the general suitable habitats for *C. cicadae*, *M. pieli*, and *P. kaempferi*: (a) SSP126-2030s; (b) SSP126-2050s; (c) SSP126-2070s; (d) SSP370-2030s; (e) SSP370-2050s; (f) SSP370-2070s; (g) SSP585-2030s; (h) SSP585-2050s; (i) SSP585-2070s; (j) provinces containing the suitable habitats.

SSP126

SSP585

SSP370

**
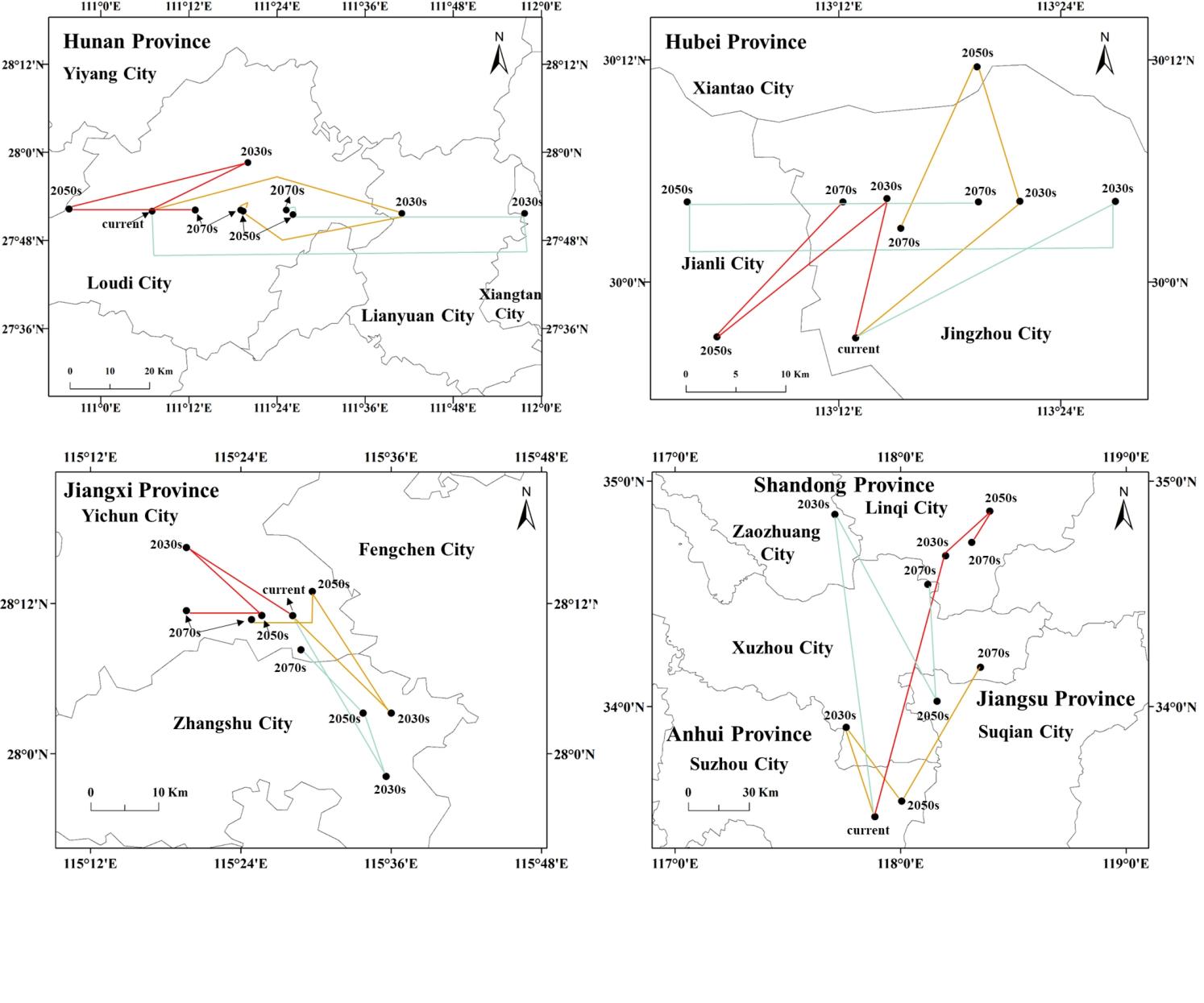
**

**Figure S8** **(A-D)** The centroids of suitable habitats under current and future climate scenarios: (A) the centroids in the general suitable habitats for *M. pieli*; (B) the centroids in the general suitable habitats for *P. kaempferi*; (C) current centroid in moderately and highly-suitable habitats for *M. pieli*; (D) centroids under future climate scenarios in moderately and highly-suitable habitats for *P. kaempferi*.

| **Species/Period** | the general suitable habitats | | moderately and highly-suitable habitats | |
| --- | --- | --- | --- | --- |
|  | **Coordinates**  (Longitude, Latitude) | **elevation** | **Coordinates**  (Longitude, Latitude) | **elevation** |
| *C. cicadae* SSP126-2030s | 110.398, 27.597 | 444m | 114.72, 28.5631 | 274m |
| *C. cicadae* SSP126-2050s | 110.269, 27.8647 | 421m | 114.495, 28.7828 | 247m |
| *C. cicadae* SSP126-2070s | 110.235, 27.8995 | 255m | 113.881, 28.5994 | 415m |
| *C. cicadae* SSP370-2030s | 110.544, 27.7545 | 332m | 115.16, 28.7399 | 120m |
| *C. cicadae* SSP370-2050s | 110.684, 27.8365 | 533m | 114.594, 28.921 | 195m |
| *C. cicadae* SSP370-2070s | 110.267, 27.7555 | 626m | 113.979, 28.7936 | 167m |
| *C. cicadae* SSP585-2030s | 110.571, 27.828 | 321m | 115.332, 28.7866 | 116m |
| *C. cicadae* SSP585-2050s | 110.792, 27.8706 | 776m | 115.155, 28.6051 | 198m |
| *C. cicadae* SSP585-2070s | 110.795, 27.8541 | 954m | 114.83, 28.6376 | 280m |
| *C. cicadae* current | 110.354, 27.7479 | 197m | 120.543, 24.1559 | 12m |
| *M. pieli* SSP126-2030s | 111.963, 27.8614 | 317m | 115.594, 27.9698 | 46m |
| *M. pieli* SSP126-2050s | 111.437, 27.8588 | 298m | 115.563, 28.0541 | 29m |
| *M. pieli* SSP126-2070s | 111.421, 27.8691 | 477m | 115.48, 28.1384 | 37m |
| *M. pieli* SSP370-2030s | 111.335, 27.9786 | 219m | 115.328, 28.3746 | 26m |
| *M. pieli* SSP370-2050s | 110.929, 27.8714 | 469m | 115.428, 28.184 | 33m |
| *M. pieli* SSP370-2070s | 111.215, 27.8691 | 379m | 115.328, 28.1904 | 56m |
| *M. pieli* SSP585-2030s | 111.6844, 27.8611 | 723m | 115.6, 28.0541 | 30m |
| *M. pieli* SSP585-2050s | 111.324, 27.8666 | 193m | 115.495, 28.2161 | 47m |
| *M. pieli* SSP585-2070s | 111.318, 27.8691 | 180m | 115.414, 28.1786 | 34m |
| *M. pieli* current | 111.117, 27.8666 | 253m | 115.469, 28.1838 | 42m |
| *P. kaempferi* SSP126-2030s | 113.449, 30.0726 | 26m | 117.709, 34.8526 | 73m |
| *P. kaempferi* SSP126-2050s | 113.064, 30.0722 | 24m | 118.162, 34.0236 | 16m |
| *P. kaempferi* SSP126-2070s | 113.326, 30.0722 | 26m | 118.121, 34.5419 | 37m |
| *P. kaempferi* SSP370-2030s | 113.244. 30.0751 | 23m | 118.2, 34.6694 | 40m |
| *P. kaempferi* SSP370-2050s | 113.091, 29.9506 | 28m | 118.396, 34.8675 | 53m |
| *P. kaempferi* SSP370-2070s | 113.204, 30.0722 | 25m | 118.316, 34.7286 | 40m |
| *P. kaempferi* SSP585-2030s | 113.363, 30.0728 | 26m | 117.759, 33.9075 | 22m |
| *P. kaempferi* SSP585-2050s | 113.325, 30.1938 | 25m | 118.005,33.5802 | 23m |
| *P. kaempferi* SSP585-2070s | 113.256, 30.0484 | 24m | 118.355, 34.174 | 60m |
| *P. kaempferi* current | 113.215, 29.9498 | 23m | 117.886, 33.5107 | 17m |

**Table S5 The coordinates and elevation of all centroids**

The following was a list of references regarding the distribution points of the *C. cicadae*, *P. kaempferi* and *M. pieli* (Chen et al., 1990, 2015, 2019, 2021; Lei et al., 1997, 2017; Zhang et al., 1998, 2017, 2021; Feng, 2002; Liu et al., 2007, 2024; Pan et al., 2011; Hu et al., 2020, 2023;Xie et al., 2016; Sun et al., 2017; Zeng et al., 2017; Huang et al., 2017; Long et al., 2017; Cui and Wei, 2018; Shi et al., 2018; Li et al., 2018, 2023; Luo et al., 2018; Xing et al., 2018; Ge et al., 2019; Huang et al., 2021; Rao et al., 2021; Shi et al., 2022; Man et al., 2022; Chen and Zhang, 2022).

References

Cui, B., and Wei, C. (2018). Ultrastructure of spermatozoa in three cicada species from China (Hemiptera, Cicadomorpha, Cicadidae). *ZooKeys*, 61–80. doi: 10.3897/zookeys.776.26966

Huang, A., Wu, T., Wu, X., Zhang, B., Shen, Y., Wang, S., et al. (2021). Analysis of Internal and External Microorganism Community of Wild Cicada Flowers and Identification of the Predominant *Cordyceps cicadae* Fungus. *Front. Microbiol.* 12. doi: 10.3389/fmicb.2021.752791

Shi, C., Song, W., Gao, J., Yan, S., Guo, C., and Zhang, T. (2022). Enhanced production of cordycepic acid from *Cordyceps cicadae* isolated from a wild environment. *Braz. J. Microbiol.* 53, 673–688. doi: 10.1007/s42770-022-00687-4

Sun, Y., Wink, M., Wang, P., Lu, H., Zhao, H., Liu, H., et al. (2017). Biological characteristics, bioactive components and antineoplastic properties of sporoderm-broken spores from wild *Cordyceps cicadae*. *Phytomedicine Int. J. Phytother. Phytopharm.* 36, 217–228. doi: 10.1016/j.phymed.2017.10.004

Feng, L. (2002). Study on Shanghai Tianma Hill *Cordyceps Sobolifera*. *J. Technol*. 125–127. doi: 10.3969/j.issn.1671-7333.2002.02.013

Liu, C., Tian, X., Tang, S., Yang, C., and Han, L. (2024). Biological Characteristics and Domestication of a Wild *Cordyceps chanhua*. *Chin J. Tropical Crops.* 45, 973–982. doi: 10.3969/j.issn.1000-2561.2024.05.012

Liu, A., Zou, X., Zhao, J., Liang, Z., Tan, A, and Zheng, Q. (2007). Biological Diversity of *Paecilomyces cicadae* Ⅰ. Morphological diversity of cicadae flower and *Paecilomyces cicadae*. *Guizhou Agric. Sci.* 9-11+161. doi: 10.3969/j.issn.1001-3601.2007.02.002

Shi, X., Zhang, H., Liu, T., Liu, Z., and Shi, X. (2018). Analysis on the diversity of endophytic fungus and identification of strain isolation in Chanhua. *China J*. *Tradit. Chin. Med*. *Pharm*. 33, 4074–4078. doi: CNKI:SUN:BXYY.0.2018-09-095

Zhang, Y., Liu, A., and Liang, Z. (1998). The formation and regeneration of protoplasts in *Isaria cicadae*. *Guizhou Agric. Sci.* 2–5. doi: CNKI:SUN:GATE.0.1998-05-000

Zhang, J., Lu, J., Zhao, X., Kang, C., Wu, Z., Deng, C., et al. (2021). The preliminary report on the species diversity of *Cordyceps* in the Doupeng Mountain region of Guizhou. *Acta Edulis Fungi*. 28, 174–187. doi: 10.16488/j.cnki.1005-9873.2021.06.022

Zhang, H., Wu, C., Yang, D., Zuo, R., Huang, W., and Chen, Y. (2017). Determination and optimal extracted conditions of adenosine in *Isaria cicadae* mycelium from different areas by ultrasonic－microwave synergistic extraction method. *Sci. Technol. Food Ind.* 38, 291-295+302. doi: 10.13386/j.issn1002-0306.2017.10.047

Zeng, W., Chang, C., Li, J., Wang, Y., Dai, Y., and Yu, H. (2017). Micro-morphology variants of *Cordyceps cicadae*. *Acta Microbiol. Sin.* 57, 350–362. doi: 10.13343/j.cnki.wsxb.20160174

Li, J., Zhang, T., and Zeng, W. (2018). Morphological Variation Pattern in Populations of *Isaria cicadae*. *Edible Fungi China*. 37, 64–69. doi: 10.13629/j.cnki.53-1054.2018.03.017

Li, H., Zheng, J., and Wang, D. (2023). Study on the natural distribution and habitat factors of *Cordyceps cicadae* in Sichuan Province. *J. Northwest For. Univ* 38, 180–184. doi: 10.3969/j.issn.1001-7461.2023.02.25

Man, H., Wang, J., Tang, G., Gui, Y., Han, J., and Zhao, J. (2022). Population Distribution Characteristics of Mating Type Gene *MAT* in *Isaria cicadae*. *Guizhou Agric. Sci.* 50, 90–98. doi: 10.3969/j.issn.1001-3601.2022.09.013

Pan, J., Jiang, F., Zheng, J., and Lu, B. (2011). Observations on the occurrence and damage characteristics of the pest *Platypleura kaempferi* in orange orchards. *S. China Fruits* 40, 39–40. doi: 10.13938/j.issn.1007-1431.2011.02.006

Luo, J., Zhang, X., Xu, R., Ren, Q., Tang, W., Ding, Z., et al. (2018). Comparison of ergosterol, cordycepin, adenosine and polysaccharide with different preparation methods in *Isaria cicadae* Miquel. *J. Anhui Agric. Univ.* 45, 389–394. doi: 10.13610/j.cnki.1672-352x.20180620.008

Hu, W., Lu, J., Deng, C., Liu, L., Meng, Q., and Fu, S (2023). Isolation and identification of medicinal *Cordyceps* strains from Xishui, Zunyi. *Guizhou Sci.* 41, 7–10. doi: 10.3969/j.cnki.1003-6563.2023.01.002

Hu, L., Deng, H., Hu, Y., Ding, H., Liu, J., Chen, Q., et al. (2020). Preliminary Study on Entomogenous Fungi in Wuyishan City of Fujian Province. *Edible Fungi China* 39, 9–13. doi: 10.13629/j.cnki.53-1054.2020.11.002

Ge, Q., Wan, J., Zhu, Y., Wang, Y., He, X., Wei, Y., et al. (2019). Qualitative and quantitative analysis of nucleoside components in *Cordyceps cicadae* by LC-MS and HPLC. *NatProd Res Dev* 31, 1857-1863+1927. doi: 10.16333/j.1001-6880.2019.11.002

Xie, C., Cao, Z., Fan, J., Xu, J., and Jia, J. (2016). The optimization of cultivation conditions for liquid strains of wild *Cordyceps cicadae*. *North. Hortic.* 165–169. doi:10.11937/bfyy.201622041

Xing, K., Shi, P., He, Z., Liu, Y., and Chen, S. (2018). Separation and Identification of the Strain from *Cordyceps cicadae* and Determination of the Nutrient Content of *Cordyceps cicadae*. *J. Anhui Agric. Sci.* 46, 162-163+171. doi: 10.13989/j.cnki.0517-6611.2018.28.048

Chen, M., Lu, L., Lin, Y., Liu, Y., and Xie, J. (2021). Study on genetic heterogeneity of *Isaria cicadae* from different sources. *J. Anhui Agric. Univ.* 48, 452–457. doi: 10.13610/j.cnki.1672-352x.20210706.020

Chen, C., Xu, J., Sun, X., Jia, J., and Gui, Z. (2015). The isolation of *Cordyceps cicadae* from the Maoshan region and the study of their polysaccharide bioactivities. *Jiangsu Agric. Sci.* 43, 347–352. doi: 10.15889/j.issn.1002-1302.2015.04.123

Chen, Z., Huang, J., Xu, Y., and Yao, L. (1990). The application of *Cordyceps cicadae* for controlling Pieris rapae. *Chin. J. Biol. Control*. 131–133. doi: 10.16409/j.cnki.2095-039x.1990.03.011

Chen, Z., and Zhang, Y. (2022). The diversity of entomopathogenic fungi species infecting similar pests in Gaoligong Mountain. *J. Baoshan Univ.* 41, 1–5. doi:10.3969/j. issn. 1674-9340. 2022.02.001

Chen, Z., Wang, Y., Dai, Y., Chen, K., Xu, L., and He, Q. (2019). Species diversity and seasonal fluctuation of entomogenous fungi of Ascomycota in Taibaoshan Forest Park in western Yunnan. *Biodivers. Sci.* 27, 993–1001. doi: 10.17520/biods.2019135

Lei, Z., Jiang, J., Li, L., and Zhou, Y. (1997). The courtship calls of *Platypleura kaempferi* from different regions. *Acta Entomol. Sin*. 349–357. doi: 10.16380/j.kcxb.1997.04.003

Lei, B., Kang, J., He, J., Wen, T., Qian, Y., and He, J. (2017). Static liquid fermentative conditions for producing N6‐(2‐hydroxyethyl) adenosine of *Paecilomyces cicadae*. *Mycosystema.* 36, 1415–1426. doi: 10.13346/j.mycosystema.170027

Rao, Y., Lang, M., Jiang, H., Tong, X., and Wang, Y. (2021). The isolation and substrate cultivation of *Cordyceps cicadae* from Tianmu Mountain in Zhejiang. *J. Zhejiang Agric Sci.* 62, 1097–1099. doi: 10.16178/j.issn.0528-9017.20210613

Huang, X., Xie, Z., Xu, J., and Zhang, B. (2017). The isolation of *Cordyceps cicadae* and the optimization of its liquid fermentation medium. *Jiangsu Agric. Sci.* 45, 153–155. doi: 10.15889/j.issn.1002-1302.2017.22.041

Long, L., Huang, S., Shi, Z., Lin, Q., Li, C., and Ding, S. (2017). Molecular Identification and Productivity on Hyaluronic Acid in Submerged Culture of Different *Cordyceps cicadae* strain. *Fujian J. Agric. Sci.* 32, 1257–1262. doi: 10.19303/j.issn.1008-0384.2017.011.017
